# Antimicrobial and Anticancer Application of Silver(I) Dipeptide Complexes

**DOI:** 10.3390/molecules26216335

**Published:** 2021-10-20

**Authors:** Gabriela Kuzderová, Michaela Rendošová, Róbert Gyepes, Simona Sovová, Danica Sabolová, Mária Vilková, Petra Olejníková, Ivana Bačová, Simonida Stokič, Martin Kello, Zuzana Vargová

**Affiliations:** 1 Department of Inorganic Chemistry, P. J. Šafárik University, Moyzesova 11, 041 54 Kosice, Slovakia; gabriela.kuzderova@student.upjs.sk (G.K.); michaela.rendosova@upjs.sk (M.R.); 2 Department of Inorganic Chemistry, Charles University, Hlavova 2030, 128 00 Praque, Czech Republic; gyepes@natur.cuni.cz; 3 Department of Biochemistry, P. J. Šafárik University, Moyzesova 11, 041 54 Kosice, Slovakia; simona.sovova@student.upjs.sk (S.S.); danica.sabolova@upjs.sk (D.S.); 4 NMR Laboratory, P. J. Šafárik University, Moyzesova 11, 041 54 Kosice, Slovakia; maria.vilkova@upjs.sk; 5 Department of Biochemistry and Microbiology, Slovak University of Technology, Radlinského 9, 812 37 Bratislava, Slovakia; petra.olejnikova@stuba.sk (P.O.); ivana.bacova@stuba.sk (I.B.); simonida.stokic@stuba.sk (S.S.); 6 Department of Pharmacology, P. J. Šafárik University, Trieda SNP 1, 040 11 Kosice, Slovakia; martin.kello@upjs.sk

**Keywords:** silver(I) complexes, dipeptide, crystal structure, stability, antimicrobial activity, anticancer activity, DNA interaction, topoisomerase I inhibition, cell cycle arrest

## Abstract

Three silver(I) dipeptide complexes [Ag(GlyGly)]*_n_*(NO_3_)*_n_* (AgGlyGly), [Ag_2_(GlyAla)(NO_3_)_2_]*_n_* (AgGlyAla) and [Ag_2_(HGlyAsp)(NO_3_)]*_n_* (AgGlyAsp) were prepared, investigated and characterized by vibrational spectroscopy (mid-IR), elemental and thermogravimetric analysis and mass spectrometry. For AgGlyGly, X-ray crystallography was also performed. Their stability in biological testing media was verified by time-dependent NMR measurements. Their in vitro antimicrobial activity was evaluated against selected pathogenic microorganisms. Moreover, the influence of silver(I) dipeptide complexes on microbial film formation was described. Further, the cytotoxicity of the complexes against selected cancer cells (BLM, MDA-MB-231, HeLa, HCT116, MCF-7 and Jurkat) and fibroblasts (BJ-5ta) using a colorimetric MTS assay was tested, and the selectivity index (SI) was identified. The mechanism of action of Ag(I) dipeptide complexes was elucidated and discussed by the study in terms of their binding affinity toward the CT DNA, the ability to cleave the DNA and the ability to influence numbers of cells within each cell cycle phase. The new silver(I) dipeptide complexes are able to bind into DNA by noncovalent interaction, and the topoisomerase I inhibition study showed that the studied complexes inhibit its activity at a concentration of 15 μM.

## 1. Introduction

For the second year in a row, humanity has been facing the pandemic caused by COVID-19 disease. The fact that this pandemic causes a huge loss of life is a direct impact of the activity of this strain of viruses. As healthcare has focused on addressing the current situation, many treatment processes have been delayed. Thus, it is natural that after two years, patients with various advanced infectious and cancerous diseases are beginning to appear in hospitals and outpatient clinics. This fact leads to the requirement for scientists to work intensively on the development of effective and selective antimicrobials and anticancer drugs. For this purpose, it is important to know what natural mechanisms the immune system in our organisms uses and to find approaches to help support this system.

All organisms, from bacteria to mammals, produce substances that they use to protect themselves against pathogens [1]. A vast majority of them are antimicrobial peptides (AMP) with short amino acid sequences, whose primarily function is protection against a wide spectrum of pathogens [2]. The first confirmed AMP was lysozyme, discovered by Alexander Fleming in 1922, which was found in vegetables and in animal tissues and secretions and whose bacteriolytic activity has been proven [3]. In mammals, leukin and phagocytin were the first AMPs to be isolated from rabbit leukocytes [4]. AMPs are short peptides, usually no longer than 100 amino acids, which generally do not coincide with commonly present sequences in peptides but are characterized by a net of the positive charges and a high ratio of hydrophobic functional groups [5]. They tend to create non-specific interactions with negatively charged components of phospholipids, such as phosphatidylglycerol, which are abundant in the membranes of microorganisms. This leads to an increase in membrane permeability and, subsequently, to a leakage of cytoplasmatic constituents and cell death.

Based on the above-mentioned facts, one of the ideas of how to aid the immune system in the case of acute conditions caused by infection is to combine metal ions with antimicrobial effects (especially Ag^+^, but also Zn^2+^ and Cu^2+^ ions) and effective organic ligands. The ideal approach is to join these metals with naturally occurring structures such as peptides. Based on many years of scientists’ experience, it is not easy to prepare such compounds. Many approaches are used to properly design the composition of a ligand to serve its purpose after it binds to a metal ion. To serve their purpose, it is important that they form compounds with the metal ion that, by their lipophilicity, electrostatic and hydrophobic interactions as well as their conformational flexibility, would be able to interact with membrane structures. Within a combination of silver ions (as one of the most antibacterial metal ions) and suitable ligands, *N*-heterocyclic carbenes and phosphines have so far been most frequently used [6,7]. In addition, research (along with our results) on carboxylic acid ligands and silver(I) and zinc(II) ions have made a significant contribution, clearly confirming that silver(I) complexes are more effective than zinc(II) complexes [8,9,10,11,12]. Silver(I) complexes with imidazole-based ligands show excellent activity against anaerobic microorganisms [13]. Silver(I) sulfadiazine (AgSD), as an active ingredient in broad spectrum antimicrobial ointments, is effective against a relatively wide range of bacteria (*S. aureus, E. coli, Klebsiella* sp., *Proteus* sp., and other *Enterobacteriaceae*) and some yeast species [14,15].

Recently, in our research, we have tried to focus on ligands that are components of the mentioned natural AMPs, amino acids, and ligands that are a part of biostructures or are needed by organisms for their activity, e.g., nicotinamide (Nam), pyridine carboxylates, or pyridine sulfonates. The aim of this selection was to prepare compounds with suitable physicochemical properties and minimal toxic effects, so that the organism would degrade the compound using natural metabolic pathways. We found that the silver(I) complexes AgGly, AgAla, and AgNam with ionic structure show significantly higher AMB (antimicrobial) activity than the neutral AgPhe complex, indicating that the cationic part of the structure may interact with negatively charged components of the cell membrane [16,17].

Our results, as well as the results of other research groups show that, in addition to the antimicrobial activity of silver(I) compounds, it cannot be overlooked that since about 2003, several works confirming the antitumour effect of silver(I) complexes have come out. They can be divided into five classes: Ag(I)-NHC complexes, Ag(I) -carboxylate complexes, Ag(I) -N-ligand complexes, Ag(I)-P-ligand complexes and mixed Ag(I) ligand complexes. These were tested against several tumour lines (e.g., kidney cancer [18,19,20,21,22], breast cancer MCF-7 [23,24,25], colorectal cancer (HCT 116) [20], lung cell lines A549 [26] tongue cancer (Cale27), HepG2, and renal adenocarcinoma (Ae498)) and in three murine cell lines (P815 mastocytoma, B16 melanoma, P388 leukaemia) [27,28,29]. The cytotoxicity in the above-mentioned articles was determined mainly by the MTT assay, but studies of mechanism of action (interaction with ctDNA, DNA nuclease activity, effect on topoisomerase I/II, cell cycle, mitochondrial membrane potential, lipophilicity, cell morphology studies, induction of oxidative stress) are not often performed.

In the case of our silver(I) complexes, we found that antitumour testing of silver(I) complexes based on amino acid and nicotinamide bioligands indicates that the ionic complexes AgGly, AgAla, and AgNam are more effective than AgPhe, and the mechanism of action predominantly depends on the structure of the presented ligand. While aliphatic ligand complexes interact with ctDNA by binding to the DNA groove, aromatic planar ligand complexes intercalate into ctDNA base pairs. We discovered that ionic silver(I) complexes of the amino acids valine and aspartic acid (AgVal and AgAsp) act selectively against *S. aureus* and *E. coli* pathogens and, at the same time, inhibit cell growth of selected tumour cell lines more than neutral AgPhe but less than AgGly and AgAla complexes with higher polarity [30].

With the idea to combine metal ions with peptide ligands, we became interested in metal dipeptide complexes’ physicochemical and biological properties. However, we found only five metal glycyl-glycinato (metal = Cd(II) [31,32]; Ag(I) [33]; Cu(II) [34]; and Zn(II) [32]), one Zn(II) glycyl-alaninato [35] and two metal glycyl-aspartato (metal = Zn(II), Co(II) [36]) complexes, mainly from structural point of view. In the structure of these dipeptide complexes, chelate (via C=O and NH_2_), bidentate (via COO^-^) and monodentate (via COO^-^ or NH_2_) coordination modes are repeated. The other physicochemical measurements, for example solid-state linear-dichroic IR spectroscopy, NMR and UV-VIS spectroscopy, EPR, ESI- and FAB mass spectroscopy and HPLC tandem mass spectroscopy, confirmed the composition, coordination mode and geometry of Au(III), Pt(II) and Pd(II) glycyl-glycinato complexes [37]. Unfortunately, the biological properties of GlyGly, GlyAla and GlyAsp metal complexes have not been studied, but several works with derivatives of these ligands and their complexes, such as Co(II), Ni(II) and Cu(II)–indol-glygly Schiff base complexes, indicate feasible antifungal and antibacterial activity [38]. Moreover ternary Cu(II)-GlyGly-L (where L means PyTA = 2,4-diamino-6-(2’-pyridyl)-1,3,5-triazine) and PzTA = 2,4-diamino-6-(2’-pyrazino)-1,3,5-triazine) and Zn(II)-GlyGly-L_1_ (where L_1_ = sulfasalazine) were synthetized and characterized and their DNA binding and cytostatic properties were evaluated [39,40].

Based on our above-mentioned experience, in the next step we focused on the preparation and characterization of silver(I) complexes with selected dipeptides in order to study their antimicrobial and anticancer activity, their toxicity, their selectivity as well as their possible mechanism of action at the nuclear level (DNA interactions, DNA cleavage, cell cycle arrest). In addition, we tested their effect on *S. aureus* biofilm formation.

## 2. Results and Discussion

### 2.1. Syntheses of Complexes

The compounds AgGlyGly, AgGlyAla and AgGlyAsp were isolated from an aqueous solution of AgNO_3_ and appropriate dipeptide at a 1:1 species:molar ratio of AgNO_3_: HGlyGly/HGlyAla/H_2_GlyAsp. The solution was then allowed to evaporate at ambient temperature under dark conditions. After two weeks, small greyish crystals were produced.

We attempted to change the crystallization conditions by adjusting the different molar ratios of the reagents (Ag:ligand 1:2, 2:1) and different solvent mixtures (for example, H_2_O:EtOH). It was found that neither changes in the ratios nor the solvent mixture influenced the final complex composition.

### 2.2. Description of Crystal Structures

**Figure 1:**
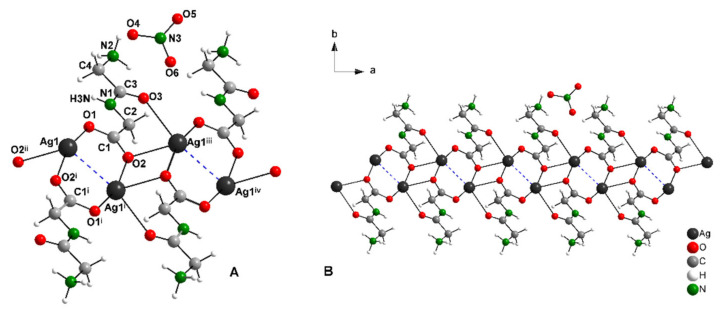
Structure motif (**A**) and solid-state packing viewed along [0 0 1] (**B**) for AgGlyGly (i = −x,−y,−z + 3; ii = x − 1,y,z; iii = x + 1,−y,2 − z).

The structural model of complex AgGlyGly is depicted in Figure 1A. Selected bond distances and angles are given in Table S2. Complex AgGlyGly crystallizes in the monoclinic lattice with space group *P2*_1_/*c* and having unit cell dimensions *a* = 4.9778(7) Å, *b* = 27.387(4) Å, *c* = 5.9015(8) Å and *β* = 94.022(2)°. The asymmetric unit consists of one molecule of HGlyGly, present as zwitterion, one silver atom and one uncoordinated nitrate molecule. Ag(I) ions are coordinated by two oxygen atoms—O1, O2—from two different molecules of HGlyGly and also by two oxygen atoms—O2, O3—from the third different molecule of HGlyGly in distorted tetrahedral geometry. Silver ions are arranged in a polymeric chain and connected by three HGlyGly molecules. The bond distance between two silver atoms Ag-Ag in the polymeric chain is 2.8930(8) Å (Figure 1A, blue dashed bonds). This bond distance is shorter than the van der Walls radii (3.44 Å), which indicates the presence of argentophilic interactions [41]. Similar argentophilic interactions were observed in complex AgAla, with Ag-Ag bond distances from 2.8722(3) Å to 3.3011(4) Å [17].

Each HGlyGly ligand in zwitterion form is coordinated to silver (I) ions through the terminal carboxylate group from one glycine residue in *syn-syn-anti* coordination mode and one molecule of ligand has tridentate properties and bridges three silver ions. The Ag—O bond distances of *syn-syn* coordinated oxygen atoms are 2.2056(39) (for Ag1–O1) and 2.1979(41) Å (for Ag1i–O2). The longer Ag–O distance of the *anti*-coordinated oxygen atom O2 is 2.5949(39) Å. Similar bond distances were observed in complex AgAla, with Ag—O bond distances of *syn-syn* coordinated oxygen atoms around 2.25 Å, and Ag—O bond distances of the *anti*-coordinated oxygen atoms around 2.65 Å. Ligands are also coordinated to silver ions by the oxygen atom O3 from the carboxylate group that belongs to the second glycine residue in monodentate coordination mode. The Ag1iii–O3 bond distance is 2.6095(39) Å. 

It is clear that coordination modes of the ligands HGlyGly led to the formation of 1D polymeric chains propagating along the *a* crystallographic axis (Figure 1B).

The search from the CSD [42] database shows one silver(I) complex with co-coordinated oxygen atoms of carboxylate groups from two ligand molecules to silver (I) ions. However, in the case of this complex, the polydentate coordination mode was not observed [33]. Zn(II), Cu(II) or Co(III) complexes were also observed with various coordination modes of dipeptide ligand HGlyGly. The most common coordination mode was bidentate bridging, which was observed by Wen-Long et al. [43]. In the case of several complexes the N,O-chelating [44] coordination mode was also found.

### 2.3. IR Spectra

To confirm the presence of organic ligands in the prepared samples, the mid-IR spectra of ligands (HGlyGly, HGlyAla and H_2_GlyAsp) and their silver(I) complexes, obtained by the ATR technique, were recorded (Figure S4), and characteristic absorption bands of these compounds are noted in Table 1.

### 2.4. Thermogravimetric Analysis

**Figure 2:**
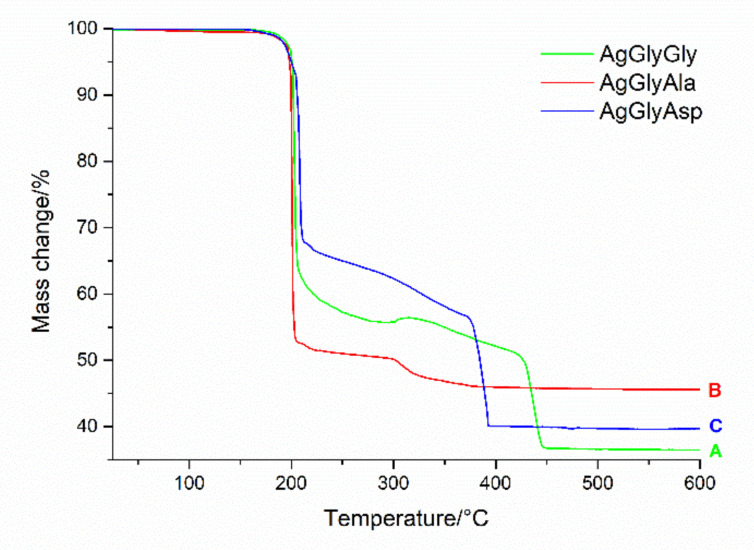
Thermogravimetric curves for studied silver(I) complexes measured in the air atmosphere in the temperature range of 25–600 °C.

TG curve of AgGlyGly, AgGlyAla and AgGlyAsp complexes are shown in Figure 2. All complexes are thermally stable to 195 °C. The thermal decompositions of the complexes inorganic-organic parts (HGlyGly/HGlyAla/H_2_GlyAsp and NO_3_^-^ ions) are observed in the range 195–400 °C, with an experimental weight loss of 63.50% (clcd.64.28%) for complex AgGlyGly (Figure 2 (green line, A)), 54.41% (clcd. 55.59%) for complex AgGlyAla (Figure 2 (red line, B)) and 59.97% (clcd. 53.79%) for complex AgGlyAsp (Figure 2 (blue line, C)). The final decomposition product is elemental silver (exp. 36.50%, clcd. 35.72% (AgGlyGly)); (exp. 45.59%, clcd. 44.41% (AgGlyAla)); and exp. 40.58%, clcd. 46.21% (AgGlyAsp)). Higher thermal stability was observed in the case of complex [CuL(H_2_O)]NO_3_ (L = GlyGly) with decomposition of the ligand GlyGly (after release of water) at 210 °C [38].

### 2.5. Stability Study

The stability of prepared compounds in aqueous solution was verified by ^1^H NMR measurements in time scale 96 h (Figure S5A–C). The chemical shift values of signals are noted in Table 2. The prepared silver(I) compounds were found to be stable in 1% DMSO-d_6_ for 96 h.

### 2.6. Antimicrobial Activity

**Figure 3:**
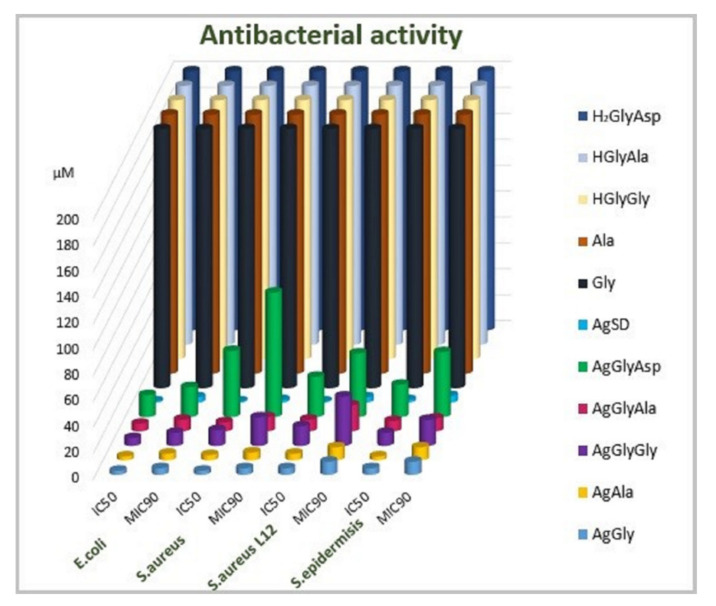
Antibacterial activity of silver(I) complex and free ligands (IC_50_, MIC_90_: µmol·L^−1^).

**Figure 4:**
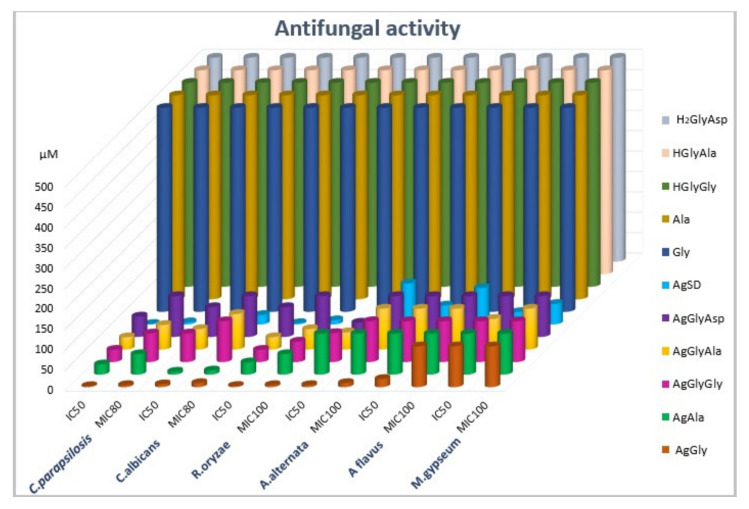
Antifungal activity of silver(I) complexes and free ligands (IC_50_, MIC_90_: µmol·L^−1^).

As mentioned in the introduction, the antimicrobial activity of silver(I) dipeptide complexes was assayed. The antimicrobial activity was compared to silver(I) complexes with single amino acid as ligands. Results for antibacterial activity are presented in Figure 3 and Table S3, and for antifungal activity in Figure 4 and Table S4.

Since all the compounds had bacteriostatic activity on the microbial cells, the inhibitory concentration MIC_90_ was evaluated. Each complex was able to inhibit bacterial cells up to 100% (data not shown), but there was a wide interval of concentration, which showed 90% cell inhibition, so we aimed to express the antibacterial activity of the evaluated complexes as the MIC_90_ values; this is the minimal concentration of the complex that is able to inhibit the growth of the cells on 90% when compared with the control (1% DMSO). As Table S3 clearly indicates, all the bacterial strains included in this study were sensitive to silver(I) complexes. In the group of model microbes, we included resistant clinical isolate *S. aureus* L12 as well. This isolate is resistant against methicillin and other clinically important antibiotics, as is mentioned in Materials and Methods. Sensitivities of all the bacterial strains in this study were comparable (Table S3). The best antibacterial activity was detected in the presence of the silver(I) complex with dipeptide GlyAla as ligand in its structure. The MIC_90_ value varied between 9 µmol·L^−1^ (*E. coli*) to 20 µmol·L^−1^ (*S. aureus* L12, the resistant strain). The antibacterial activity of dipeptide complexes decreased in the order AgGlyAla > AgGlyGly > AgGlyAsp.

It seems that the results of antibacterial activity reflect the transport activity of dipeptides. Peptide transporters are important membrane proteins, enabling the transport of nutrients for nitrogen metabolism. Thus, microbes are able to synthesize amino acids for proteosynthesis, often using the protein residues—the short peptides—from the environment. Even where there are more transporter proteins with similar substrate specificity, the malfunction of these transporters leads to the decreased viability of microbial cells. Therefore, these transporters could be presented as a new attractive target size for antimicrobial therapy. Peptide transporters are usually used for the transport of peptide analogues with antimicrobial activity or for compounds that are able to compete with the natural substrate, making the peptide residues unavailable for the cell. In our work, the complexes were designed as silver(I) complexes with dipeptides ligands, where the ligands serve as delivery systems for the active Ag^+^ ion with many targets in microbial cells. In the intracellular acidic pH of cytoplasm, the complexes are decomposed. Amino acids could be incorporated in the bacterial metabolism, and silver ions could act antibacterially. The dipeptide transporters were most studied in gram negative bacteria *E. coli* and *S.* Thyphimurium. These bacterial cells have three permeases for peptide transport: the oligopeptide, tripeptide and dipeptide permeases [45]. Thus, these permeases have an overlapping substrate specificity; the explanation of antimicrobial activity seems to be the substrate specificity of dipeptide permease (Dpp). The name of the permease implies a preference for dipeptide substrates, but it can also transport tripeptides to a lesser extent [46,47]. Overall, Dpp has a broad substrate specificity, but it is less tolerant to side-chain modification [48] and has a stricter requirement for a free C-terminal *a*-carboxyl group.

**Figure 5:**
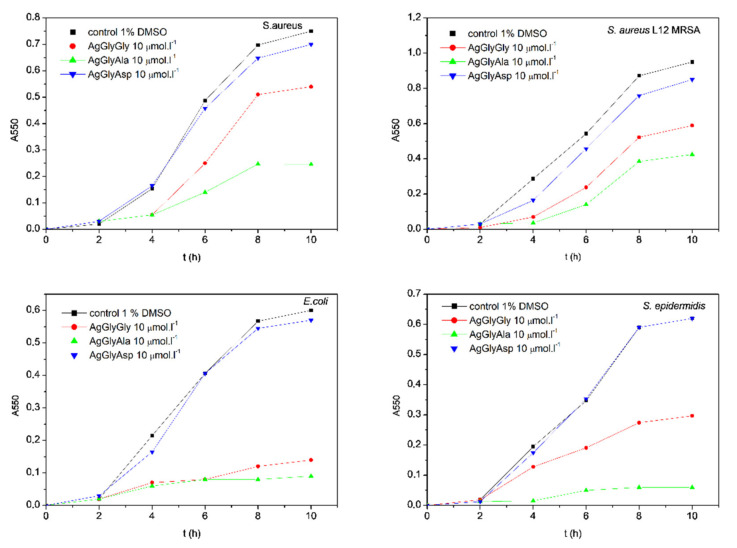
Growth of bacterial cells in the presence of silver(I) complex after addition to the lag phase.

**Figure 6:**
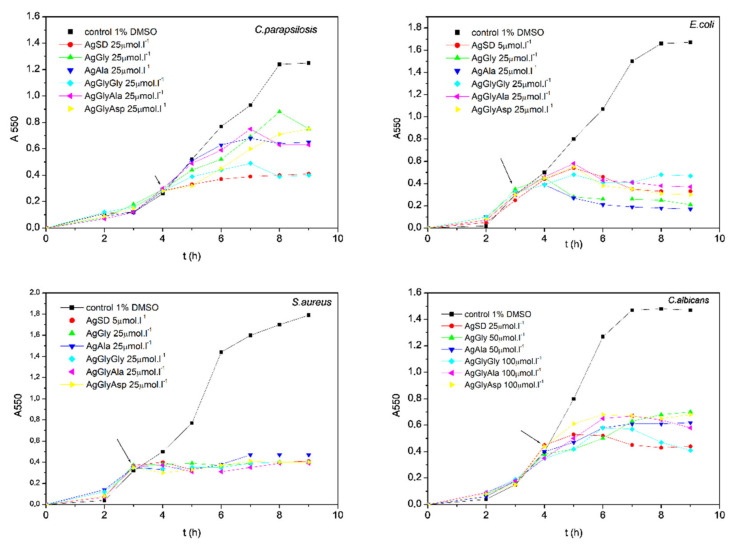
The growth inhibition after addition of the silver(I) complexes in the early exponential growth phase.

The antibacterial activity of these complexes probably depends on their bioavailability—in this case, the substrate binding properties and the specificity of mentioned dipeptide permease. As was published by Smith et al. (1999) [47] the affinity of the DppA transporter of *E. coli* varied for the substrates. The results showed that the highest affinity of the transporter for dipeptides that had the same first amino acid in dipeptide order (in our case Gly) was dependent on the second one in dipeptide order. The highest affinity of the transporter was described for alanine (X-Ala), followed by the dipeptide with glycine as the second amino acid (X-Gly). The transporter had the lowest affinity for the dipeptide, where the second amino acid was aspartic acid (X-Asp) [47]. The affinity of the transporter most probably explains the activity of our complexes. Our results point to the fact that the antibacterial activity of diamino acid complexes with silver correlates with the affinity of the transporter. The AgGlyAla complex was most effective, followed by the AgGlyGly complex, while the AgGlyAsp complex inhibited the growth of Gram-negative bacteria to the lowest extent. Similar antimicrobial activity was observed in model staphylococci assayed in our study. We assayed the antimicrobial potential of newly synthesized silver(I) complexes when these were added to the cells at the beginning of the lag phase and the exponential phase, when the cells have the highest growth rate. As is shown on Figure 5 and Figure 6, complexes were able to inhibit the growth of bacteria and yeasts in both cases.

We assayed the activity of these complexes on the bacterial biofilm. As is described in Table 3, the staphylococcal biofilm formation capability decreased. The formation of yeast biofilm was not influenced by the tested complexes; therefore, the data are not shown directly. In the presence of silver(I) complexes, the biofilm formed by *Candida parapsilosis* was identical to the control conditions. The highest antifungal activity was observed in the presence of the AgGlyAla complex (Table S4). The growth of all model filamentous fungi was reduced in the presence of the AgGlyAla complex. We suppose that the antimicrobial activity of dipeptide complexes is the interplay between the bioavailability and the silver amount (%) in the complex; the higher the silver content, the higher antimicrobial activity. Finally, we tested all the mentioned complexes in this work for their mutagenic activity using the Ames test on *S. thypimurium* TA98 and *S. thyphimurium* TA100, detecting the frameshift with respect to point mutations. None of the tested complexes demonstrated mutagenic activity, since the number of revertants was comparable to that observed for the negative control and spontaneous revertants (Table 4). Considering all our obtained results, such types of complexes have a great potential in the design of new antimicrobial, especially antibacterial, active structures.

### 2.7. Anticancer Activity

In addition to antimicrobial activity, we also tested silver(I) complexes with dipeptides against selected cancer cell lines. In contrast to previously selected cancer lines [30], we also used breast cancer lines for these complexes to find out how effective our complexes are against these two cancer lines (MCF-7-model for hormone therapy, MDA-MB-231-model for chemotherapy). The results are presented as IC_50_ in Table 5. It is clear from the results that the complexes have a similar effect on colon HCT116 and cervical HeLa cancer cell lines, and these are similar to commercially used cisplatin (cisPt), except for colon HCT116 where cisPt is almost four times more active than AgGlyAla and almost five times more active than AgGlyGly and AgGlyAsp. However, the situation is different for breast double negative MCF-7 and breast triple negative MDA-MB-231 cancer cell lines. In the case of MCF-7 cells, the AgGlyGly and AgGlyAla complexes are even more effective (AgGlyGly three times, IC_50_ = 9.0 µM, AgGlyAla four times, IC_50_ = 7.5 µM) than cisPt (IC_50_ = 29.7 µM), and in the case of MDA-MB-231, the AgGlyAla complex has the same activity (IC_50_ = 7.4 µM for complex, IC_50_ = 7.1 µM for cisPt). A similar effect to that against MDA-MB-231 was observed for the human leukemic T cell lymphoma cancer line Jurkat (see Table 5). Against BLM-type melanoma, AgGlyGly and AgGlyAla complexes are less active than cisPt but more active than against colon HCT116 cancer cells. The AgGlyAsp complex is least effective against all cell lines tested with similar IC_50_ values.

Based on the observed results, the silver(I) dipeptide complex activity can be noted in the following order: AgGlyAla ˃ AgGlyGly ˃ AgGlyAsp.

Comparing the silver(I) dipeptide complex activity of MCF-7 and MDA-MB-231 (different models for therapy), a similar effect can be seen in the IC_50_ values. A similar observation was presented by Christina N. Banti et al. [49]; their ([Ag_3_(Gly)_2_NO_3_]*_n_*) complex provided the same IC_50_ values against MCF-7 and MDA-MB-231 (2.6 ± 0.1 and 2.7 ± 0.2 μM, respectively). In contrast, Türkan Kutlu et al. [50] observed slightly different IC_50_ values for both cancer cell lines in the case of three silver(I) carbene complexes (18, 17, and 23 μM for MCF-7 and 8, 7.5, and 12 μM for MDA-MB-231).

**Figure 7:**
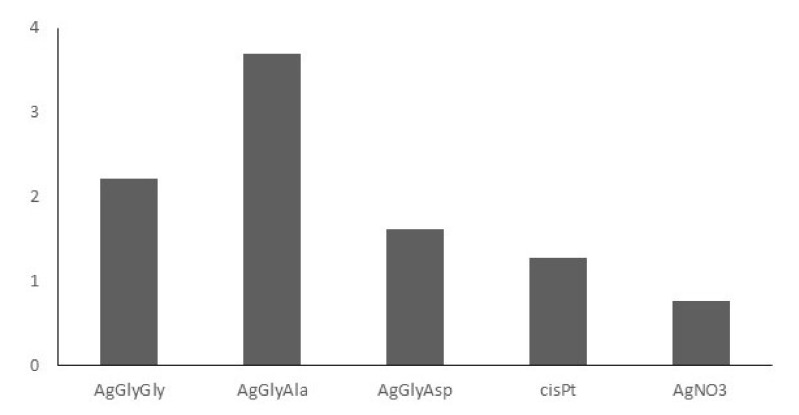
Selectivity index (SI) of Ag(I) dipeptide complexes for BJ-5ta/BLM.

The selectivity index (SI), calculated as the ratio of IC_50_ values for specific normal and cancer cell lines of skin tissue, is depicted on Figure 7. It is evident that the silver(I) dipeptide complexes’ selectivity reproduces the complexes’ activity order, so AgGlyAla reveals the highest selectivity (SI = 3.7).

### 2.8. DNA Binding Properties

#### 2.8.1. UV-Vis Spectroscopy

Absorption spectroscopy is one of the most useful methods to study the binding of any drug to DNA. “Hyperchromic” and “hypochromic” effects are the spectra features of DNA concerning its double helical structure. The extent of hypochromism generally indicates the intercalative or groove binding strength [51]. On the contrary, the hyperchromic effect might be ascribed to external contact (electrostatic binding) or to partial uncoiling of the helix structure of DNA, exposing more bases of the DNA [52].

**Figure 8:**
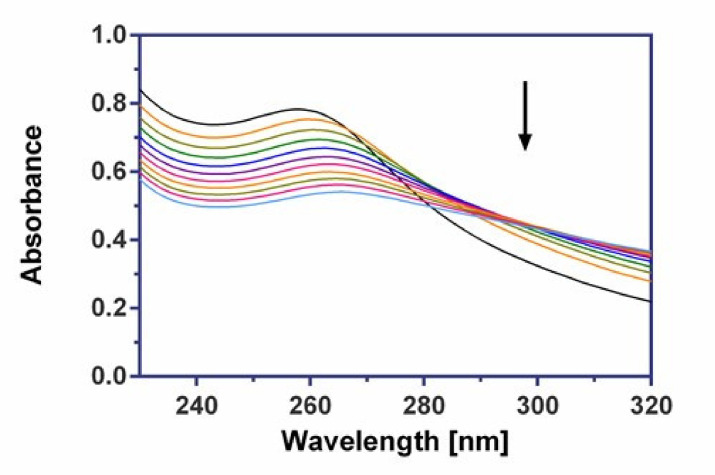
Absorption spectra of AgGlyAsp in 0.01 M Tris buffer with increasing concentration of CT DNA. The black line corresponds to the spectrum of the free complex.

Figure 8 illustrates the spectral changes in Tris-HCl solution for the AgGlyAsp complex during titration with increasing amounts of CT DNA. The initial spectrum is of a fixed concentration (7.85 × 10^−5^ M) of the free complex in the absence of CT DNA. We observed the appreciable changes in the position of the maximum wavelength on addition of CT DNA (red shift of 13 nm), and the intensity of the band of the AgGlyAsp complex at 260 nm increases, resulting in 30% hypochromism (Table 6). Spectroscopic titration of complexes AgGlyGly and AgGlyAla are shown in Supplementary Materials Figures S6 and S7.

We noticed a significant bathochromic shift for all the investigated silver(I) complexes. The values of these red shifts are given in Table 6. It was assumed that the magnitude of this shift could be interpreted as an indication of the strength of the interaction between the DNA structure and the ligand considered [53].

#### 2.8.2. Fluorescence Measurements

Fluorescence spectroscopy has extensive applications as a tool to study complex/DNA interactions due to its ability to examine the effects of environmental conditions on various DNA interactions. It is considered as a sensitive non-destructive technique for the characterization of DNA–ligand interactions [54].

**Figure 9:**
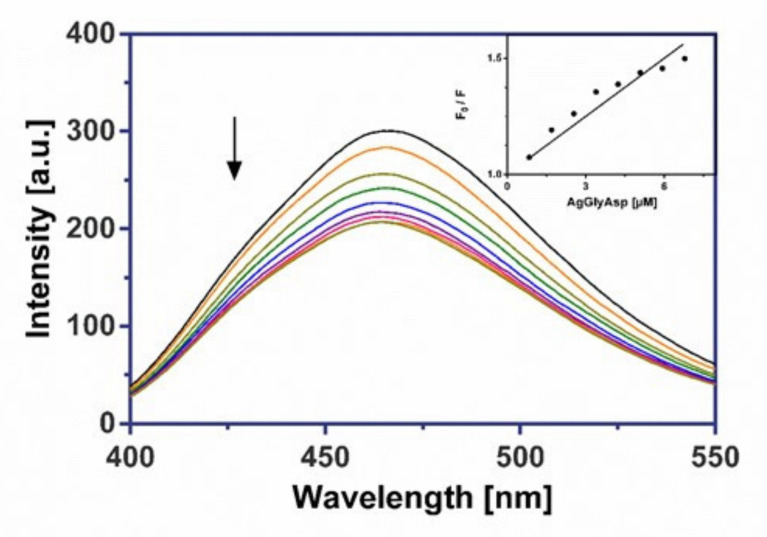
The emission spectra of CT DNA–HO in the presence of AgGlyAsp. Arrow shows the intensity changes upon increasing the complex concentration. Inset: the corresponding Stern–Volmer plot.

**Figure 10:**
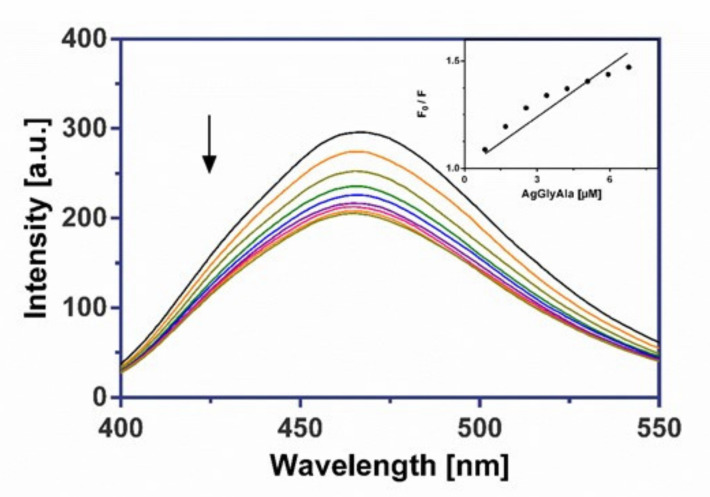
The emission spectra of CT DNA–HO in the presence of AgGlyAla. Arrow shows the intensity changes upon increasing the complex concentration. Inset: the corresponding Stern–Volmer plot.

**Figure 11:**
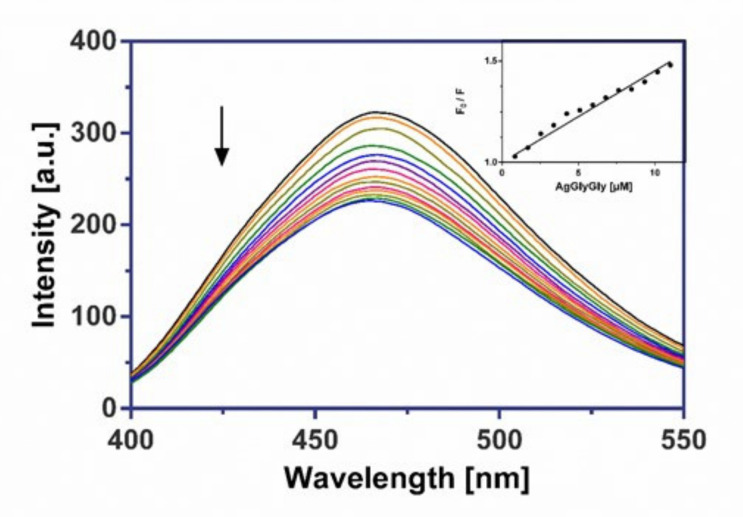
The emission spectra of CT DNA–HO in the presence of AgGlyGly. Arrow shows the intensity changes upon increasing the complex concentration. Inset: the corresponding Stern–Volmer plot.

The investigated silver(I) dipeptide complexes did not exhibit fluorescence at room temperature in solution or in the presence of CT DNA, and their binding to DNA could not be directly predicted through the emission spectra. Because our complexes don’t include an aromatic skeleton, we assumed they would not be intercalators like Ethidium bromide. Hence, a competitive binding study of each complex with frequently used groove binder Hoechst 33258 (HO) was carried out to understand whether the complex could displace HO from its CT DNA–HO system. Therefore, a comparison of the intrinsic emission spectra of the free DNA–HO system in presence of the silver(I) complex can be useful in evaluating the mode of DNA interaction with the complexes. In this experiment, the successive addition of three investigated Ag(I) complexes to the DNA–HO system resulted in a marked decrease in the fluorescence maximum at 462 nm (Figure 9, Figure 10 and Figure 11).

The considerable displacement of Hoechst 33258 by synthesized complexes confirmed the groove-binding mode for all drugs. The values of the quenching constants (Ksv), obtained from the fluorescence emission spectra of the CT DNA-HO system in the absence or in the presence of increasing amounts of Ag(I) complexes, are given in Table 6 and imply that complex AgGlyAsp shows a moderately greater ability to release the groove binder Hoechst 33258 from DNA than AgGlyGly and AgGlyAla. The calculated Stern-Volmer constants (Ksv) are slightly higher than for earlier researched silver(I) glycinate and aminoacidate complexes [16,17].

### 2.9. CD Spectroscopy

Circular dichroism (CD) spectroscopy is an optical technique that measures the difference in the absorption of left and right circularly polarized light. This technique has been widely employed in the study of nucleic acid structures, and the use of it to monitor the conformational polymorphism of DNA has grown tremendously in the past few decades [55].

**Figure 12:**
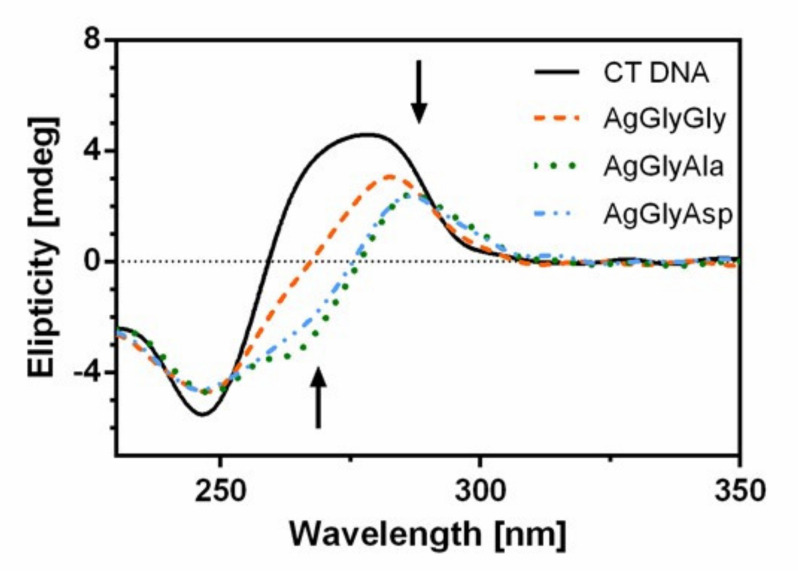
CD spectra of CT DNA in 10 mM Tris buffer in the presence of silver(I) dipeptide complexes.

To gain insight into the complex-DNA interaction and, in particular, into the conformational changes of DNA double helix induced by the silver(I) dipeptide complexes, CD spectroscopy was used. CT DNA, a model of right-handed DNA-duplex structure (B-form), shows two characteristic bands consisting of a positive one at 275 nm due to base stacking and a negative one at 245 nm due to the right-handed helicity of DNA [56]. In general, intercalation of the classical DNA intercalator increases the intensities of both CD bands due to stabilization of the double helical conformation of B-DNA, whereas non-intercalation modes such as electrostatic interactions and groove bindings of small molecules show a decrease or no perturbation in base stacking and helicity bands [17]. The circular dichroic spectra of CT DNA in the absence and presence of the Ag(I) complexes are given in Figure 12.

We noticed a red shift as well as a decrease in the intensity of the positive band and increase of the negative CD band. This result indicates the groove-binding nature of the investigated silver(I) dipeptide complexes. Previously, we recorded a similar course of the CD spectrum for the bioactive glycinate [16] and aminoacidate complexes [17].

### 2.10. Viscosimetry

The interaction between the metal complexes and DNA was further confirmed by viscometric measurements. The viscosity of DNA, which is sensitive enough to DNA length changes, can offer convincing evidence for the interactive binding mode of complexes with DNA. In general, classical intercalative binding viscosity will rise. In contrast, a partial and/or non-intercalative binding could bend the DNA helix, causing a reduction of its valid length, with the DNA viscosity staying invariant or displaying a slight reduction [57,58].

**Figure 13:**
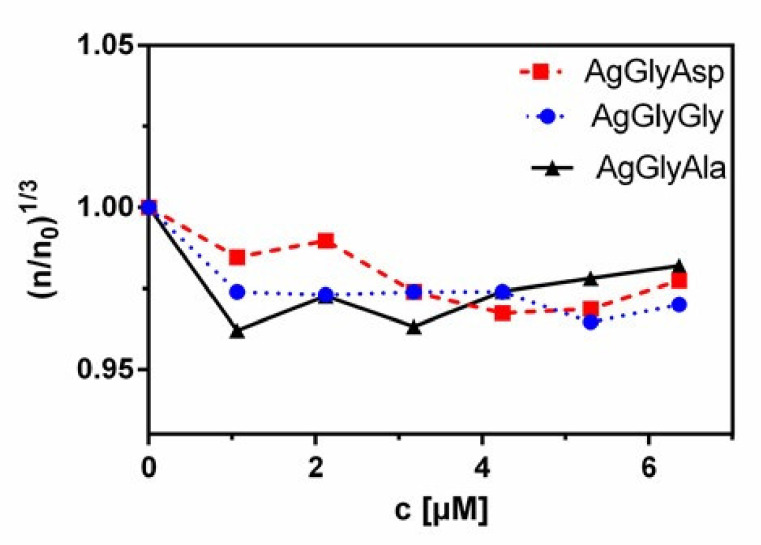
Effect of increasing amounts of silver(I) dipeptide complexes on the viscosity of CT DNA in 10 mM Tris buffer.

We found that the addition of the silver(I) dipeptide complexes caused a small decrease in the initial viscosity, indicating the groove-binding mode of all three Ag(I) complexes (Figure 13). The analogous viscosimetric values were established for the [Cu(dtb)(phen)]^2+^ complex, which binds into the minor groove of DNA [59].

### 2.11. Topoisomerase I Inhibition

Human topoisomerases (Topo) are ubiquitous enzymes that catalyse the topological changes of DNA during replication, transcription, recombination, repair, chromatin assembly and chromosome segregation by triggering, breaking and re-joining of the DNA strand [60]. Many studies have confirmed DNA topoisomerases as therapeutic targets in cancer chemotherapy [61]. The topoisomerase inhibitory activity is directly related to the conversion of supercoiled DNA to its relaxed form [62].

**Figure 14:**
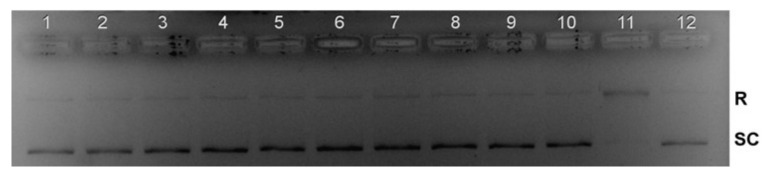
The inhibition of human Topo I by different concentrations of silver(I) dipeptide complexes. Lanes 1–3: pBR322 + Topo I + AgGlyGly; Lanes 4–6: pBR322 + Topo I + AgGlyAla; Lanes 7–9: pBR322 + Topo I + AgGlyAsp, Lane 10: pBR322; Lane 11: pBR322 + Topo I; Lane 12: pBR322 + Topo I + camptothecin. SC: supercoiled DNA, R: relaxed DNA.

To establish the mechanism of anticancer action, a standard plasmid (pBR322) cleavage assay was used to study the effect of Ag(I) complexes on Topo I by DNA gel electrophoresis. Agarose gel electrophoresis (Figure 14) shows the effect of drugs (Lanes 1–3: AgGlyGly: 15, 30, 60 μM; Lanes 4–6: AgGlyAla: 15, 30, 60 μM; Lanes 7–9: AgGlyAsp: 15, 30, 60 μM) on the relaxation of supercoiled DNA (SC) by topoisomerase I.

The monitored relaxation activity indicates that all three complexes inhibit the human Topo I activity at 15 μM concentrations. The inhibition activity of Topo I was observed at a lower concentration than the previously investigated silver(I) pyridine-2-sulfonate complex [63].

### 2.12. Cell Cycle Analysis

**Figure 15:**
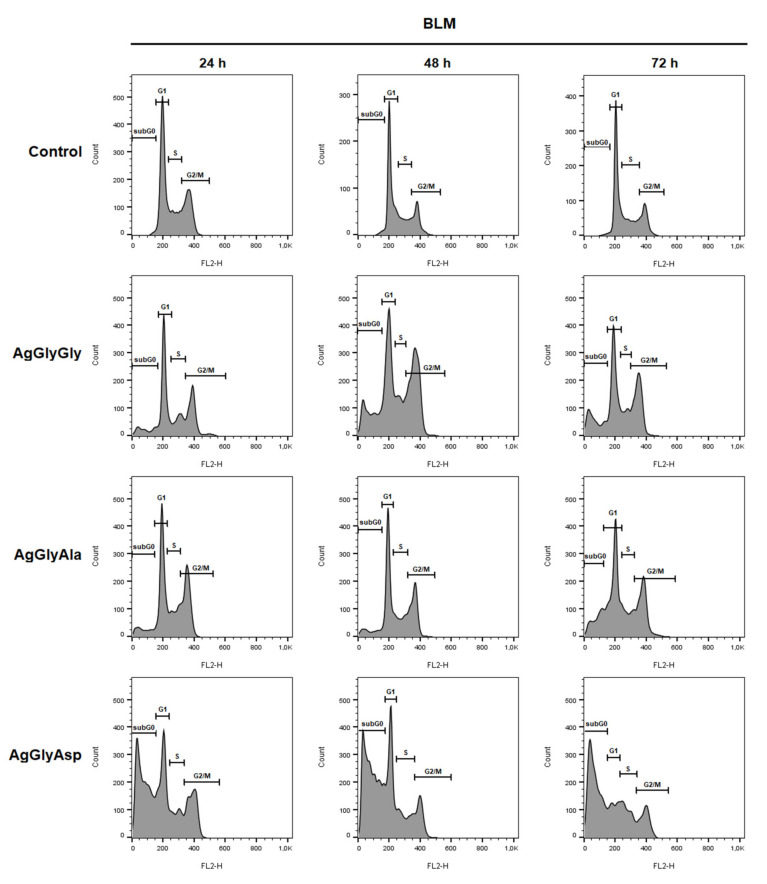
Histogram representation of cell cycle distribution in BLM cells treated with IC50 AgGlyGly, AgGlyAla and AgGlyAsp after 24 h, 48 h and 72 h. Representative data of three independent experiments are presented.

With the aim of elucidating the mechanism of anticancer activity of silver(I) dipeptide complexes, the changes in cell cycle were studied. The cell cycle distribution changes of BLM, HCT116, Jurkat and MDA-MB-231 cells were analysed after treatment with IC_50_ of AgGlyGly, AgGlyAla and AgGlyAsp complexes by flow cytometry after 24, 48 and 72 h incubation time (Tables S5–S8, Figure 15 and Figures S8–S10).

The untreated BLM cells (Control) were spread 49.2% in the G1 phase, 18.6% in the S phase and 30.1% in the G2/M phase after 24 h incubation with an additional 2.2% of cells in sub G0 subpopulation (marker of apoptosis). A similar percentage of cells in the cell cycle phases was observed for Control groups after 48 and 72 h incubation. As shown in Figure 15, in treated BLM cells, AgGlyGly and AgGlyAla complexes influence mainly the number of cells in the G1 (decrease) and G2/M (increase) phase of cell cycle after all incubation timepoints as compared to the Control. In the case of complex AgGlyAsp, a significant increase in the number of apoptotic cells in the sub-G0 population (51.7% after 24 h, 56.1% after 48 and 59.3% after 72 h) was observed compared to the Control group (2.2, 2.0, 1.6%).

The untreated HCT cells (Control) were spread 0.8% in the sub-G0 population, 50.9% in the G1 phase, 18.5% in the S phase and 29.8% in the G2/M phase after 24 h incubation. The number of cells in sub-G0 population and in G1 cell cycle phases was increased after 48 and 72 h incubation, and in S and G2/M phases, it remained approximately the same (20.0% after 48 h in S phase) or decreased in all remaining phases. As shown in Figure S8, in treated HCT cells, all complexes influenced the number of cells in the sub-G0 population (increase), G1 (decrease) and G2/M (decrease) phase of cell cycle after all incubation timepoints as compared to the untreated Control. The proportion range of apoptotic cells in the sub-G0 population was from 32.4 to 44.3% after 24 h, from 46.2 to 57.1% after 48 h and from 56.8 to 62.9% after 72 h incubation for treated cells with silver(I) dipeptide complexes.

A similar trend was observed in the case of the cell number as compared to the untreated and treated Jurkat cells with silver(I) dipeptide complexes (Figure S9).

However, in the case of MDA-MB-231 cells, the diversification of Control cell population and cells treated with AgGlyGly through cell cycle phases remained approximately the same after all incubation times (Figure S10 and Table S8). In the case of AgGlyAla and AgGlyAsp the number of cells in the G1 phase decreased, similarly to the previous cancer cells. On the other side, the number of cells in the G2/M phase increased in the case of both complexes, from 18.6% (Control) to 22.8% (AgGlyAla) or 30.6% (AgGlyAsp) after 24 h and from 15.3% to 19.4% (AgGlyAla) or 25.8% (AgGlyAsp). After 72 h of treatment, the number of cells in this phase was again decreased, with a concomitant increase of the sub-G0 cell population due to the pro-apoptotic effect of the tested compounds.

To compare our observations, we found research groups that investigated the cell cycle effect of silver(I) complexes in sub-G1, G0/G1, S or G2/M phases for MCF-7 and MDA-MB-231 cells. While in the case of silver(I) complexes with diclofenac and niflumic acid [25] and NSAID drugs (aspH, salH_2_, napH, pHbzaH, tpAs) [64], results showed the increase of apoptotic cells in the sub-G1 phase, in the case of NSAID drugs of nimesulide and derivatives of pnictogen, cell proliferation was predominantly suppressed by inhibiting DNA synthesis and including S-phase cell cycle arrest [23]. On the other hand, [Ag(phen)(L)]NO_3_ complex {phen = 1,10-phenantroline; L = 2-formylpyridine-N(4)-R-thiosemicarbazone} induced accumulation of MDA-MB-231 cells in sub-G1, which indicates internucleosomal DNA fragmentation [65]. In addition, the cell cycle analysis of silver(I) thiosulfate complex (STS) showed that the MCF-7 cell population at the G1 phase significantly increased (from 45.3% (Control) to 57.4% after 12 μM (STS) and 56.2% after 24 μM (STS)), suggesting that STS arrests the cell cycle of MCF-7 at the G1 phase [66]. In the case of series three Ag(I)-NHC complexes, all compounds induced apoptosis of MCF-7 and MDA-MB-231 cancer cells with the cell cycle arrest in the G1 phase (IC_50_ and 24 h incubation were used) [50]. Banti et al., inter alia, presented the influence of [Ag_3_(Gly)_2_NO_3_]*_n_* complex on the progression of MFC-7 cancer cell cycles. Comparing the percentage of cells in all cell cycle phases, an increase was observed in the sub-G0 population (from 9.8% for the Control to 42.7% for treated cells) and in the S phase (from 13.2% for the Control to 24.1% for treated cells), so cell cycle arrest and apoptosis occurrence was confirmed.

## 3. Materials and Methods

### 3.1. Materials

Glycyl-*L*-Glycine (HGlyGly) was purchased from Fluka (Switzerland); Glycyl-*L*-Alanine (HGlyAla) from Roanal (Hungary); Glycyl-*L*-Aspartic acid (H_2_GlyAsp); calf thymus DNA (CT DNA), Hoechst 33258, plasmid DNA (pBR322), tris(hydroxymethyl)aminomethane (Tris), propidium iodide (PI), phosphate buffered saline (PBS), agarose and dimethyl sulfoxide (DMSO) were purchased from Sigma-Aldrich Chemie (Germany); ethidium bromide (EB) from Boehringer Mannheim (Germany); topoisomerase I from Takara (Japan); cisplatin from Acros Organics (USA); and silver(I) nitrate from Lachema (Czech Republic). Topoisomerase I was purchased from Inspiralis. Other chemicals were analytically pure and used without purification.

### 3.2. Synthesis

**Preparation of [Ag(GlyGly)]_n_(NO_3_)_n_ (AgGlyGly)**. Glycyl-*L*-Glycine (69.9 mg, 0.53 mmol) was dissolved in water (6 mL) and was added to 5 mL of silver(I) nitrate aqueous solution (100 mg; 0.53 mmol). The reaction mixture was stirred for 10 min and then allowed to stand in the absence of light. Crystals formed by slow evaporation (after 2 weeks) were filtered off, dried in the air and used for further characterisation. Yield (based on AgNO_3_): 35%. Elemental analysis (C_4_H_8_Ag_1_N_3_O_6_): Calcd (%) C 15.90; H 2.67; N 13.91; Found: C 16.10; H 2.60; N 13.83. MS(+) *m*/*z*: 106.98; 149.95; 586.87 (see Figure S1 in ESI).

**Preparation of [Ag_2_(GlyAla)(NO_3_)_2_]_n_ (AgGlyAla)**. Glycyl-*L*-Alanine (77.3 mg, 0.53 mmol) was dissolved in water (6 mL) and was added to 5 mL of silver(I) nitrate aqueous solution (100 mg; 0.53 mmol). The reaction mixture was stirred for 15 min and then allowed to stand in the absence of light. Crystals formed by slow evaporation (after 2 weeks) were filtered off, dried in the air and used for further characterisation. Yield (based on AgNO_3_): 44%. Elemental analysis (C_5_H_10_Ag_2_N_4_O_9_): Calcd (%) C 12.35; H 2.07; N 11.53; Found: C 11.5; H 1.99; N 11.0. MS(+) *m*/*z*: 248.30; 566.34; 604.34 (see Figure S2 in ESI).

**Preparation of [Ag_2_(HGlyAsp)(NO_3_)]_n_ (AgGlyAsp)** Glycyl-*L*-Aspartic acid (110 mg; 0.58 mmol) was dissolved in deionized water (10 mL) and was added dropwise to 4 mL of silver(I) nitrate aqueous solution (100 mg; 0.53 mmol). The reaction mixture was stirred constantly for 15 min and then allowed to stand at ambient temperature in the absence of light. Crystals formed by slow evaporation (after 2 weeks) were filtered off, dried in the air and used for further characterisation. Yield (based on AgNO_3_): 36%. Elemental analysis (C_6_H_9_Ag_2_N_3_O_8_): Calcd (%) C 15.44; H 1.94; N 9.00; Found: C 15.32; H 1.87; N 8.47. MS(+) *m*/*z*: 106.9; 147.9; 252.9 (see Figure S3 in ESI).

### 3.3. Physical Measurements

Single-crystal diffraction data for complex AgGlyGly were collected on a Bruker D8 VENTURE Kappa Duo PHOTON 100 diffractometer using Mo Kα radiation (λ = 0.71073 Å). The sample specimens were cooled to 120 K using an Oxford Cryostream cooler. Experimental data were processed by diffractometer software. The phase problem was solved by intrinsic phasing, and the structure models were refined against F2 using the SHELX program suite [67]. All non-hydrogen atoms were refined anisotropically. Hydrogen atoms were put into idealized positions and were refined isotropically. Crystal data are listed in Table S1. The structure figures were drawn using the DIAMOND software [68]. Single-crystal diffraction data were collected for complex AgGlyAsp. However, only the complex structural model was proposed because, despite repeated efforts to prepare more suitable crystals, all experiments failed.

Infrared spectra were recorded on a Nicolet Avatar FT-IR 6700 (Fourier transform infrared spectroscopy) spectrometer from 4000 to 400 cm^−1^ using the ATR (attenuated total reflectance) technique.

*Elemental analysis* was performed with a CHNOS Elemental Analyzer Vario MICRO from Elementar Analysensysteme GmbH.

The thermal behaviour of compounds AgGlyGly, AgGlyAla and AgGlyAsp was studied by thermogravimetry (TG) using Setaram Setsys Evolution analyser-1750 in the atmosphere of air. Samples were heated with a heating rate of 10 °C·min^−1^ in the temperature range from 25 to 600 °C and with an air flow rate of 60 cm^3^·min^−1^. Before thermal measurements, gentle grinding of the sample and careful packing into the corundum crucibles were performed. The mass of samples used in the analyses was within 4–10 mg. Obtained thermoanalytical curves were analysed using the Origin computational program (version 6.1052, Origin Lab Northampton, MA, USA).

The mass spectrum was recorded on Waters Acquity QDa mass spectrometer in a positive mode in the range of 50 *m*/*z* to 1200 *m*/*z*. Both AgGlyGly and AgGlyAla were dissolved in water. AgGlyAsp was dissolved in methanol of HPLC quality. Identification of the molecular species was based on theoretical monoisotopic mass values.

Nuclear resonance data were collected on a Varian VNMRS 600 spectrometer operating at 599.87 MHz for ^1^H. The concentration of all samples was approximately 3 mg/0.6 mL of 1% DMSO d_6_/D_2_O. The chemical shifts were referenced to the TSP (3- (trimethylsilyl)propionic-2,2,3,3-d4 acid sodium salt) peak (^1^H NMR 0.00 ppm). Before NMR experiments, freshly prepared complex samples were diluted, ^1^H NMR experiments were performed and the complex samples were kept in the dark for 24, 48, 72 and 96 h and the measurements were repeated.

### 3.4. Antimicrobial Activity of Newly Prepared Complexes Assayed In Vitro

The antimicrobial activities of silver(I) complexes AgGly, AgAla, AgGlyGly, AgGlyAla and AgGlyAsp and free ligands (HGly, HAla, HGlyGly, HGlyAla and H_2_GlyAsp) were evaluated by the macrodilution method [69,70] using G^+^ bacteria (*Firmicutes*) *Staphylococcus epidermidis* CCM 7221 *Staphylococcus aureus* CCM 3953 (Czech Collection of Microorganisms), methicillin-resistant *Staphylococcus aureus* L12 (MRSA; clinical isolate from central venous catheter, resistant against penicillin, methicillin, cefoxitin, erythromycin, chloramphenicol and ciprofloxacine; *MecA* gene confirmed) G^−^ bacteria (*γ-proteobacteria*) *Escherichia coli* (*E. coli*) CCM 3988 (Czech Collection of Microorganisms) and the yeasts *Candida parapsilosis* ATCC 22019 (American type culture collection) and *Candida albicans* SC 5314 (Candida strains genome database). Cultures of bacteria (in Mueller-Hinton broth, MHB) and yeasts (in Sabouraud’s growth medium, SB) were incubated under shaking (250 rpm) at 37 °C. The growth of bacteria and yeasts was evaluated by measuring the absorbance of the growing cultures (A550) until the cultures reached the stationary growth phase. The effects of silver(I) complexes on the growth of filamentous fungi *Rhizopus oryzae* CCM F-8284, *Alternaria alternata* CCM F-128 and *Microsporum gypseum* CCM F-8342 were observed by the macrodilution method on solidified potato-dextrose growth medium (PDA). During culturing, the diameters of growing fungal colonies were measured at regular intervals [71,72]. Briefly, the antimicrobial activity (bacteria and yeasts) was assayed by two approaches: silver(I) complexes/ligands were added to the microbial culture at the beginning of the cultivation-to the lag phase (I) or silver(I) complexes/ligands were added to the microbial cultures in the exponential phase of growth (II). Chromatographically pure silver(I) complexes/ligands were dissolved in DMSO. The final concentration of DMSO never exceeded 1.0 vol. % in the control or treated samples. The concentration of silver(I) complexes used in experimental work for the evaluation of antimicrobial activity was used in the range of 0.1-100 µmol·L^−1^, and for the free ligands in the range of 0.5–2.0 mmol·L^−1^ in all experiments. The antimicrobial activity of tested compounds was characterized by IC_50_ values (concentration of a compound that inhibits the growth of model microorganism by 50% when compared to the untreated control) and also by MIC_90_ values (minimal concentration of a compound that inhibits the microbial growth by 90%); for yeasts the MIC_80_ (minimal concentration of a compound that inhibits the microbial growth by 80%) was evaluated. The IC_50_ and MIC_90_ with respect to MIC_80_ values were evaluated from toxicity curves. After addition of the active complexes into the exponential growth phase, the minimal concentration of the active compounds that was able to block the growth of the culture was evaluated. All the obtained results of antimicrobial activity were compared to the activity of AgSD (silver(I) sulfadiazine), the silver(I) complex used in clinical practice.

#### 3.4.1. Assessment of Potential Mutagenic Activity

Potential mutagenic activity of silver(I) complexes AgGly, AgAla, AgGlyGly, AgGlyAla, AgGlyAsp and AgSD were assayed by the plate incorporation method [73], without metabolic activation, using *Salmonella* Typhimurium TA98 CCM 3811 and *Salmonella* Typhimurium TA100 CCM 3812. *Salmonella* Typhimurium TA 98 and TA 100 were obtained from the Czech Collection of Microorganisms. As a positive mutagen, 3-(5-nitro-2-furyl) acrylic acid (NFAA) was used. As a positive response, the mutagenic activity of the silver(I) complexes was defined as a reproducible two-fold increase of revertants with a dose-response relationship and statistical evaluation using the t-test.

#### 3.4.2. Influence of Silver Complexes on Microbial Biofilm Formation

The influence of silver(I) complexes AgGly, AgAla, AgGlyGly, AgGlyAla, AgGlyAsp and AgSD were tested for their biofilm eradication capability. The biofilm was qualified by the crystal violet staining method. Briefly, overnight cultures of bacteria *S.aureus* (in MHB) and yeasts *C.parapsislosis* (in SB) were prepared. The fresh prepared growth media MHB and SB were inoculated with the overnight culture for a final concentration of 5 × 106 cells mL^−1^. The microbial biofilm was formed in 96-well microplates for adherent cells. A total of 198 μL of inoculated MHB and SB was added to 2 μL of silver(I) complex (100–0.1 µmol·L ^−1^). The formation of biofilm was influenced by silver(I) compounds for the whole period of biofilm formation. Prepared cultures were incubated for 2 h at 37 °C. After the adherence phase (2 h), the growth media together with non-adherent cells were removed, and fresh media and with indicated silver(I) complexes were added again to the cells that adhered on the wells of microplate. The treatment with DMSO (solvent) alone served as a control. The inoculated microplates were incubated at 37 °C for the next 46 h. The effect of silver(I) compounds on the biofilm formation was compared with the untreated control. The intensity of biofilm formation was evaluated using the crystal violet dye method [74]. Microplates with mature biofilm were decanted and washed using 200 μL of saline solution. The plate was left for 15 min to dry completely at laboratory temperature. Then, 110 μL of 0.4% crystal violet dye was added to each well, and the plate was incubated for 15 min at laboratory temperature. After the decantation, the plate was washed 3 times with 200 μL of saline solution. Then, 200 μL of 96% ethanol was added. The microplate was left to incubate for 15 min at laboratory temperature. After the transfer of the well content to a clean microplate, the absorbance (570 nm) of released crystal violet (proportionate to biofilm cell density) was measured,, and the values were evaluated as weak biofilm formation A_570_ <0.1; 0.199>, medium biofilm formation A_570_ <0.2; 0.3>, strong biofilm formation A_570_ <0.301; 0.999>, or very strong biofilm formation A_570_ ≥ 1.00.

### 3.5. Anticancer Activity

#### 3.5.1. Cell Culture

The cell lines BLM (human metastatic melanoma), HCT116 (human colorectal carcinoma), Jurkat (human leukemic T cell lymphoma) and MDA-MB-231 (human mammary gland adenocarcinoma) were obtained from (ATCC, Manassas, VA, USA). HCT, Jurkat and MDA-MB-231 cells were cultured in growth medium RPMI 1640 (Biosera, Kansas City, MO, USA) supplemented with a 10% foetal bovine serum (FBS) (Invitrogen, Carlsbad, CA, USA) and 1X HyClone™ Antibiotic/Antimycotic Solution (GE Healthcare, Piscataway, NJ, USA). BLM cells were cultured in growth medium consisting of high glucose Dulbecco’s Modified Eagle Medium (DMEM) + sodium pyruvate (Biosera) supplemented with a 10% FBS and Antibiotic/Antimycotic solution. Cells were maintained in standard conditions with an atmosphere containing 5% CO_2_ at 37 °C. Prior to each experiment, cell viability was greater than 95%.

#### 3.5.2. MTS Assay

The antiproliferative activity of Ag(I) complexes AgGlyGly, AgGlyAla and AgGlyAsp and free ligands (HGlyGly, HGlyAla and H_2_GlyAsp) were evaluated by colorimetric microculture MTS (3-(4,5-dimethylthiazol-2-yl)-5-(3-carboxymethoxyphenyl)-2-(4-sulfophenyl)-2H tetrazolium) assay (Promega, Madison, WI, USA). Cancer and non-cancer cells were seeded at a density of 5 × 10^3^ cells/well in 96-well polystyrene microplates. Twenty-four hours after cell seeding, different concentrations (1.0–100.0 µM) of the compounds were tested. After 72 h of incubation, 10 µL of MTS was added to each well. After an additional 2 h, cell proliferation was evaluated by measuring the absorbance at wavelength 490 nm using the automated Cytation™ 3 Cell Imaging Multi-Mode Reader (Biotek, Winooski, VT, USA). The absorbance of control wells was taken as 100%, and the results were expressed as a percent of the untreated control. IC_50_ values were calculated from MTS analyses.

### 3.6. DNA Binding Properties

#### 3.6.1. UV–Vis Absorption Measurements

UV–vis spectra were measured in 0.01 M Tris-HCl buffer (pH 7.4) using a Varian Cary 100 Bio spectrophotometer in the range of 230–320 nm and recorded at room temperature (24 °C) using a quartz cuvette of 1 cm path length. The concentration of investigated Ag(I) complexes was 7.85 × 10^−5^ M. During the titration, an aliquot of buffered CT DNA solution was added to each cuvette (sample and reference) to eliminate the absorbance of DNA itself. The DNA concentration per nucleotide was determined by absorption spectroscopy using the molar absorption coefficient (6600 M^−1^cm^−1^) at 260 nm.

#### 3.6.2. Fluorescence Binding Study

The fluorescence competitive binding studies of silver(I) complexes with Hoechst 33 258 (HO) were performed on a Varian Cary Eclipse spectrophotometer. The emission spectra of DNA–HO were measured in the range of 400–550 nm using an excitation wavelength of 343 nm with a slit width of 5 nm for the excitation and 5 nm for the emission beam. The DNA–HO complex was prepared by adding 1.2 μM of dye and 0.3 μM of CT DNA in 10 mM Tris-HCl buffer (pH 7.4, 24 °C). *K_sv_* quenching constant was determined according to the classical Stern-Volmer Equation (1) in [63,75]:

*F*_0_/*F* = 1 + *K_SV_* [*Q*] (1)

where *F*_0_ and *F* are the fluorescence intensities in the absence and the presence of the quencher at 462 nm, *K_SV_* is the Stern-Volmer quenching constant and [*Q*] is the concentration of the quencher.

#### 3.6.3. Circular Dichroism Measurements

The CD spectra of DNA in the presence of Ag complexes were recorded on a JASCO (J-810) spectropolarimeter in the wavelength range of 230–330 nm in 10 mM Tris-HCl buffer (pH 7.4) at 24 °C. The CD spectrum was collected after averaging over three accumulations, and the scan speed was 200 nm/min. The concentration of CT DNA was 5.4 × 10^−5^ M and concentration of compounds was 4.2 μM.

#### 3.6.4. Viscosimetry

The viscosity of the samples was measured at 24 °C using a digital Fungilab ViscoLead ONE Rotational viscometer. For solution viscosity measurements, CT DNA concentration was kept fixed at 4.1 × 10^−5^ M, while the concentration of silver(I) complexes changed from 1.06 μM to 6.36 μM. The values of relative specific viscosity (ɳ/ɳ_0_)^1/3^ were obtained by calculating the ratio of ɳ_0_ to ɳ, where ɳ is the viscosity of DNA in the absence (ɳ_0_) and in the presence of Ag(I) complex (ɳ). These values were plotted against concentration of silver(I) peptide complex [76].

#### 3.6.5. Topoisomerase I Inhibition Studies

The Topoisomerase I (Topo I) inhibition activity was determined as previously described by Smolkova et al. [77]. The reaction mixture consisted of 3 units of Topoisomerase type I, 1 unit of 0.1% bovine serum albumin, 10 units of buffer (2 μM/mixture) and 1 unit of supercoiled plasmid DNA (pBR322) in the presence of silver(I) complexes at various concentrations (15, 30 and 60 μM). Upon Topo I addition, reaction mixtures were incubated at 37 °C for 30 min. The reaction mixtures were analysed on 1% agarose gel and electrophoresed at 7 V/cm for 2 h, with TAE (Tris-acetate-EDTA) as the running buffer. After electrophoresis, the gel was stained in an EB solution (1 mg/mL) and washed in water, and DNA bands were visualized under UV light.

### 3.7. Cell Cycle Analysis

For cell cycle analyses, adherent and floating cells were harvested at three different times (24 h, 48 h and 72 h) after treatment with IC_50_ dipeptides silver(I) complexes, washed in cold PBS, fixed in cold ethanol (70%) and stored at −20 °C until analysis. Prior to each analysis, cells were washed in PBS, followed by resuspending in staining solution (0.5 mg/mL ribonuclease A, 0.2% final concentration Triton X-100, propidium iodide 0.025 mg/mL in 500 µL PBS) (all Sigma Aldrich), and incubated for 30 min at room temperature in the dark. For analysis of stained cells, a BD FACSCalibur flow cytometer (BD Bio-sciences) was used. A minimum of 1 × 10^4^ events were analysed per analysis.

#### Statistical Analysis

Results are expressed as average ± SD. Statistical analyses of the data were performed using standard procedures, with one-way ANOVA followed by the Bonferroni multiple comparisons test. Differences were considered significant when *p* < 0.05. Throughout this paper, * indicates *p* < 0.05, ** *p* < 0.01 and *** *p* < 0.001 vs. untreated control.

## 4. Conclusions

With the aim to find a feasible antimicrobial and anticancer structural model similar to AMPs, we prepared a series of silver(I) dipeptide complexes. For their composition and stability characterization, we used X-ray analysis, IR spectroscopy, elemental analysis, thermal and MS analysis and ^1^H NMR measurements. Antimicrobial activity was tested by IC50 and MIC determination using the macrodilution method, and potential mutagenic activity (toxicity) was verified by the plate incorporation method. Moreover, the influence of complexes on microbial biofilm was quantified by the crystal violet staining method. Comparing antibacterial and antifungal activity of the prepared silver(I) dipeptide complexes, complexes have a great potential mainly against bacteria. From the biofilm formation point of view, the significant ability to reduce its formation was observed in the case of complex AgGlyAla. In addition, none of the tested complexes showed mutagenic activity, because the number of revertants was comparable to the negative control and spontaneous revertants. The highest antibacterial activity was observed in the AgGlyGly and AgGlyAla complexes. It important to note the activity of tested complexes on resistant *S. aureus*. Considering the IC_50_ and MIC_90_ values, the sensitivity of resistant and sensitive strains was comparable. This fact makes these compounds very important for combatting the microbial resistance.

Anticancer testing confirms the silver(I) dipeptide complexes’ activity against selected cancer cell lines, mainly against breast double negative carcinoma MCF-7 and breast triple negative carcinoma MDA-MB-231. However, IC_50_ values of most active AgGlyAla against MCF-7 (hormone depended) and MDA-MB-231 (hormone independent) are similar (7.5 and 7.4 μM, respectively), which confirms no hormone mimetic behaviour, similar to the silver(I) glycinate complex of Christina N. Banti [49]. The results of spectroscopic and viscosimetric measurements indicate that the studied Ag(I) complexes bind to the DNA groove with moderate strength. In addition, all silver(I) dipeptide complexes exhibit Topo I inhibition activity at 15 μM concentration, resulting in their good anticancer effect.

From the point of view of complexes’ ability to influence the cell cycle, the treatment with AgGlyGly after 24 h significantly induced cell cycle arrest in the G2/M phase, with a maximum at 48 h only in BLM cells compared with untreated cells. Moreover, in all tested cell lines (except MDA-MB-231), we noticed a significant increase in the sub-G0 population of cells, with the highest peak at 72 h after AgGlyGly treatment. This population is typically considered as a population of apoptotic cells. The treatment with AgGlyAla induced a G2/M phase block in BLM (24 h) and MDA-MB-231 cells (24–48h) and significantly increased the sub-G0 population of cells at 24 h, with a maximum at 72 h. Only in BLM cells was the sub-G0 population maintained at the same increased level from 24 to 72 h. The last treatment with AgGlyAsp induced a G2/M phase block only in MDA-MB-231 cells, with maximum at 48 h, and significantly increased the sub-G0 population compared with untreated cells. In HCT116 and Jurkat cell lines, we noticed that dipeptide silver(I) complexes only significantly increased in the sub-G0 population of cells without cell cycle block. This effect was accompanied in parallel with a decrease of the cell population in the G1, S and G2/M phases. These results suggest that treatment with dipeptide silver(I) complexes contributes to cell cycle arrest in the G2/M phase and induces apoptosis in a time-dependent manner.

## Figures and Tables

**Table 1:** IR spectral data assignments for complexes AgGlyGly, AgGlyAla and AgGlyAsp and their appropriate ligands HGlyGly, HGlyAla and H_2_GlyAsp.

	HGlyGly	AgGlyGly	HGlyAla	AgGlyAla	H_2_GlyAsp	AgGlyAsp
*ν*(NH_3_^+^) + *ν*(CH_2_)	3350–2500	3350–2500	3350–2500	3350–2500	3350–2500	3350–2500
*ν*(NH_2_)	3283	3317	3199	3350	3327	3258
					3162	3072
*ν*(CH)	-	-	2813	2977	2996	2950
	-	-			2958	2907
*δ*(NH_3_^+^),*δ*(NH_2_)*_scissoring_*	1653	1645	1684	1666	1666	1682
					1512	1505
*ν*_as_(COO^−^)	1572	1522	1526	1550	1568	1515
*ν*_s_(COO^−^)	1478	1446	1406	1437	1445	1436
Δ(*ν*_as_ − *ν*_s_)		76		113		79
*δ*(CCH)	-	1396	1370	1365	1386	1368
	-	-	1348		1335	1325
*ν*(NO_3_^-^)	-	1316	-	1291	-	1325
*ν*(CCN)*_alif_*.	1096	1077	1032	1032	1041	1039
				1061	838	822
*ν*(CC)	964,916	907	935	911	937	935
	899	824	917	873,856	862	823
γ(COO^−^)	733	716	774	781,762	763	764
*δ*(CCO)	588,528	598,548	562	545	564	553

**Table 2:** ^1^H NMR data of complexes AgGlyGly, AgGlyAla and AgGlyAsp and their appropriate ligands HGlyGly, HGlyAla and H_2_GlyAsp.

Chemical Shifts δ/ppm						
Signal	HGlyGly	AgGlyGly	HGlyAla	AgGlyAla	H_2_GlyAsp	AgGlyAsp
A	3.86	3.86	4.19	4.18	4.62	4.49
B	3.83	3.83	3.83	3.82	3.88	3.82
C	-	-	1.37	1.36	2.90	2.76; 2.52

**Table 3:** The influence of silver(I) complexes on *S. aureus* biofilm formation.

	CC (µM)	AgSD		AgGly		AgAla		CC (µM)	AgGlyGly		AgGlyAla		AgGlyAsp	
		A_570_	I	A_570_	I	A_570_	I		A_570_	I	A_570_	I	A_570_	I
**S. aureus**	50	0.20	medium	0.28	medium	0.29	medium	100	0.28	medium	0.25	medium	0.25	medium
	25	0.18	medium	0.27	medium	0.29	medium	50	0.22	medium	0.23	medium	0.24	medium
	10	0.20	medium	0.37	strong	0.39	strong	25	0.26	medium	0.25	medium	0.31	medium
	5	0.18	medium	0.45	strong	0.35	strong	10	0.39	strong	0.41	strong	0.35	strong
	1	0.29	medium	0.40	strong	0.47	strong	5	0.72	strong	0.51	strong	0.36	strong
	**control** A_570_ = 0.47, I–strong													

Gly: glycine, Ala: alanine, SD: sulfadiazine, CC: complex concentration, I: biofilm intensity.

**Table 4:** Results of Ames tests.

Complex Dose per Plate	Revertants CFU No. ± SD	
	*S. thypimurium* TA100	*S. thypimurium* TA98
**AgSD**		
20 ng	Toxicity	Toxicity
10 ng	167 ± 12	30 ± 8
5 ng	165 ± 12	25 ± 7
2.5 ng	125 ± 7	35 ± 7
**AgGly**		
100 ng	Toxicity	Toxicity
40 ng	140 ± 18	32 ± 2
20 ng	155 ± 7	36 ± 3
10 ng	140 ± 14	35 ± 1
5 ng	129 ± 12	29 ± 1
**AgAla**		
100 ng	Toxicity	Toxicity
40 ng	187 ± 15	28 ± 2
20 ng	172 ± 28	38 ± 4
10 ng	157 ± 4	34 ± 1
5 ng	175 ± 7	33 ± 5
**AgGlyGly**		
200 ng	135 ± 21	36 ± 2
100 ng	138 ± 2	31 ± 1
50 ng	162 ±17	36 ± 2
20 ng	149 ± 12	29 ± 1
**AgGlyAla**		
200 ng	Toxicity	Toxicity
100 ng	115 ± 7	28 ± 2
50 ng	130 ± 14	34 ± 2
20 ng	142 ± 8	27 ± 2
	**AgGlyAsp**	
200 ng	149 ± 7	27 ± 1
100 ng	141 ± 9	22 ± 3
50 ng	129 ± 12	26 ± 3
20 ng	137 ± 10	29 ± 1
**Negative control 1%DMSO**	130 ± 5	29 ± 2
**Spontaneous revertants**	160 ± 14	28 ± 3
**Positive control** **NFAA (100µg)**	1352± 124	28 ± 2

Gly: glycine, Ala: alanine, SD: sulfadiazine, NFAA: 3-(5-nitro-2-furyl) acrylic acid.

**Table 5:** Anticancer activity of free ligands HGlyGly, HGlyAla, and H_2_GlyAsp and silver(I) dipeptide complexes (IC_50_: µM).

	HCT116	HeLa	MCF-7	MDA-MB-231	Jurkat	BLM	BJ-5ta
HGlyGly	>300	>300	>300	>300	>300	>300	>300
HGlyAla	>300	>300	>300	>300	>300	>300	>300
H_2_GlyAsp	>300	>300	>300	>300	>300	>300	>300
AgGlyGly	34.5	38.3	9.0	13.0	14.8	21.2	47.1
AgGlyAla	28.0	33.2	7.5	7.4	9.7	10.9	40.2
AgGlyAsp	35.5	33.3	33.8	33.6	36.8	35.6	57.6
cisPt	7.4	35.4	29.7	7.1	6.3	29.5	37.9
AgNO_3_	21.8	45.3	13.2	25.9	6.6	31.5	24.3

Note: HCT: human colorectal carcinoma, HeLa: human cervical adenocarcinoma, MCF-7: human breast double negative carcinoma, MDA-MB-231: human breast triple negative carcinoma, Jurkat: human leukemic T cell lymphoma, BLM: human amelanotic melanoma, BJ-5ta: human skin fibroblasts.

**Table 6:** Spectral and binding characteristics of Ag(I) complexes.

	λ_max_ (nm)	Δλ_max_ (nm)	Hypochromism (%)	*K_SV_* (M^−1^)
AgGlyAsp	260	13	30.8	8.33 ± 0.07 × 10^4^
AgGlyAla	268	9	40.7	7.97 ± 0.03 × 10^4^
AgGlyGly	264	22	20.1	4.51 ± 0.05 × 10^4^

## Data Availability

The data presented in this study are available on request from the corresponding author. The CIF data (CCDC 2116542) for AgGlyGly can also be obtained free of charge from the Cambridge Crystallographic Data Centre via http://www.ccdc.cam.ac.uk/data_request/cif (accessed on 17 October 2021).

## Supplementary Materials

The following are available online. Figure S1: IR spectra of ligand HGlyGly, HGlyAla, H_2_GlyAsp and their AgGlyGly, AgGlyAla and AgGlyAsp complexes; Figure S2A-C: ^1^H NMR spectra of HGlyGly ligand and time dependent spectra of AgGlyGly complex recorded in 1% DMSO d_6_/D_2_O (A), HGlyAla ligand and time dependent spectra of AgGlyAla complex (B) and H2GlyGly ligand and time dependent spectra of AgGlyAsp complex (C); Figure S3: Spectroscopic titration in Tris-HCl solution for the AgGlyGly complex during titration with increasing amounts of CT DNA; Figure S4: Spectroscopic titration in Tris-HCl solution for the AgGlyAla complex during titration with increasing amounts of CT DNA; Figure S5: Histogram representation of cell cycle distribution in HCT116 cells treated with IC_50_ AgGlyGly, AgGlyAla, AgGlyAsp after 24 h, 48 h, and 72 h. Representative data of three independent experiments are presented; Figure S6: Histogram representation of cell cycle distribution in Jurkat cells treated with IC_50_ AgGlyGly, AgGlyAla, AgGlyAsp after 24 h, 48 h, and 72 h. Representative data of three independent experiments are presented; Figure S7: Histogram representation of cell cycle distribution in MDA-MB-231 cells treated with IC_50_ AgGlyGly, AgGlyAla, AgGlyAsp after 24 h, 48 h, and 72 h. Representative data of three independent experiments are presented; Figure S8: Mass spectra of the aqueous solution of AgGlyGly complex measured in positive mode; Figure S9: Mass spectra of the aqueous solution of AgGlyAla complex measured in positive mode; Figure S10: Mass spectra of the methanolic solution of AgGlyAsp complex measured in positive mode; Table S1: Crystal data and structure refinement summary for complex AgGlyGly; Table S2: Selected bond distances (Å) and angles (°) for AgGlyGly; Table S3: Antibacterial activity of silver complex and free ligands (IC_50_, MIC_90_: µmol.l^-1^); Table S4: Antifungal activity of silver complexes and free ligands; Table S5: Cell cycle analysis of BLM cells after 24 h, 48 h, and 72 h incubation with dipeptides complexes; Table S6: Cell cycle analysis of HCT116 cells after 24 h, 48 h, and 72 h incubation with dipeptides complexes; Table S7: Cell cycle analysis of Jurkat cells after 24 h, 48 h, and 72 h incubation with dipeptides complexes; Table S8: Cell cycle analysis of MDA-MB-231 cells after 24 h, 48 h, and 72 h incubation with dipeptides complexes.

## Sample Availability

Samples of the compounds AgGlyGly, AgGlyAla and AgGlyAsp are available from the authors.
