# Supplementary material for: Antimicrobial and Anticancer Application of Silver(I) Dipeptide Complexes
Source: Molecules. 2021 Oct 20;26(21):6335. doi: 10.3390/molecules26216335 (PMC8587849; doi:10.3390/molecules26216335)
Supplement: Supplementary file 1 [file molecules-26-06335-s001.zip › molecules-1425715-supplementary.pdf]

Supplementary material

A strong perspective on antimicrobial and anticancer application of silver(I) dipeptide complexes

Gabriela Kuzderová[1], Michaela Rendošová[1], Róbert Gyepes[2], Simona Sovová[3], Danica Sabolová[3], Mária Vilková[4], Petra Olejníková[5], Ivana Bačová[5], Simonida Stokič[5], Martin Kello[6], Zuzana Vargová[1]*

[1]*Department of Inorganic Chemistry, P. J. Šafárik University, Moyzesova 11, 041 54 Kosice, Slovakia*

[2]*Department of Inorganic Chemistry, Charles University, Hlavova 2030, 128 00 Praque, Czech Republic*

[3]*Department of Biochemistry, P. J. Šafárik University, Moyzesova 11, 041 54 Kosice, Slovakia*

[4]*NMR Laboratory, P. J. Šafárik University, Moyzesova 11, 041 54 Kosice, Slovakia*

[5]*Department of Biochemistry and Microbiology, Slovak University of Technology, Radlinského 9, 812 37 Bratislava, Slovak Republic*

[6]*Department of Pharmacology, Faculty of Medicine, P. J. Šafárik University, Trieda SNP 1, 04011 Košice, Slovak Republic*

Figure S1. IR spectra of ligand HGlyGly, HGlyAla, H_2GlyAsp and their AgGlyGly, AgGlyAla and AgGlyAsp complexes.

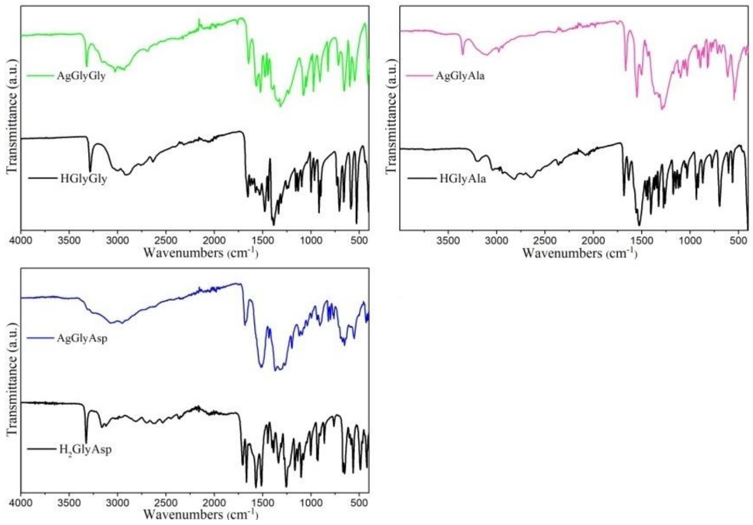

Figure S2A-C. ^{1}H NMR spectra of HGlyGly ligand and time dependent spectra of AgGlyGly complex recorded in 1% DMSO d_6/D_2O (A), HGlyAla ligand and time dependent spectra of AgGlyAla complex (B) and H_2GlyGly ligand and time dependent spectra of AgGlyAsp complex (C).

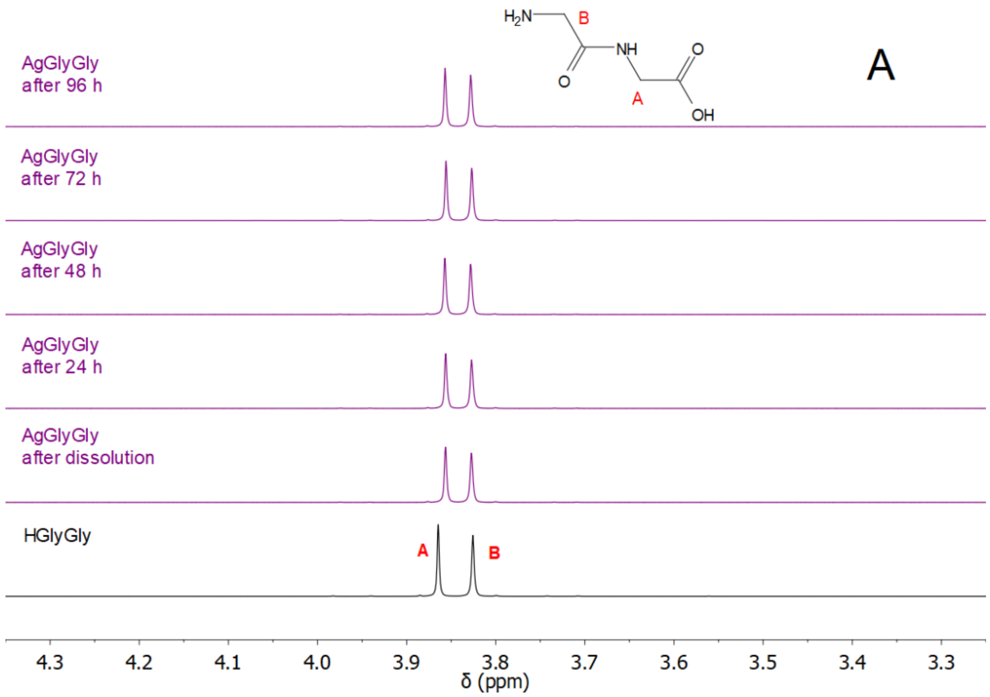

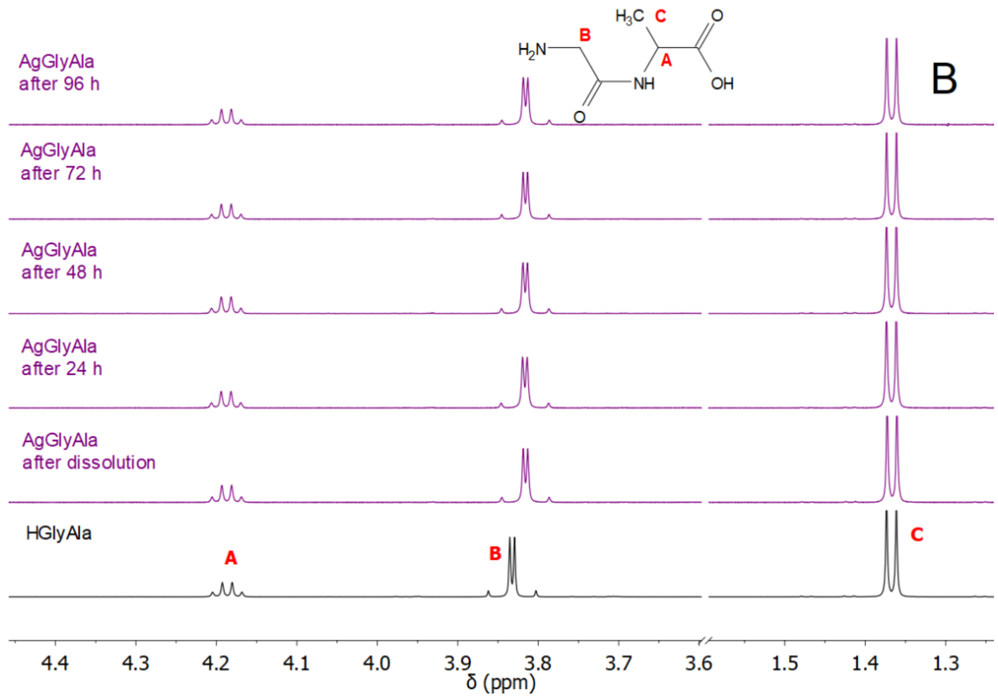
B
AgGlyAla
after 96 h
AgGlyAla
after 72 h
AgGlyAla
after 48 h
AgGlyAla
after 24 h
AgGlyAla
after dissolution
HGlyAla
A
B
C
H2N
H3C
NH
OH
O
4.4
4.3
4.2
4.1
4.0
3.9
3.8
3.7
3.6
1.5
1.4
1.3
δ (ppm)

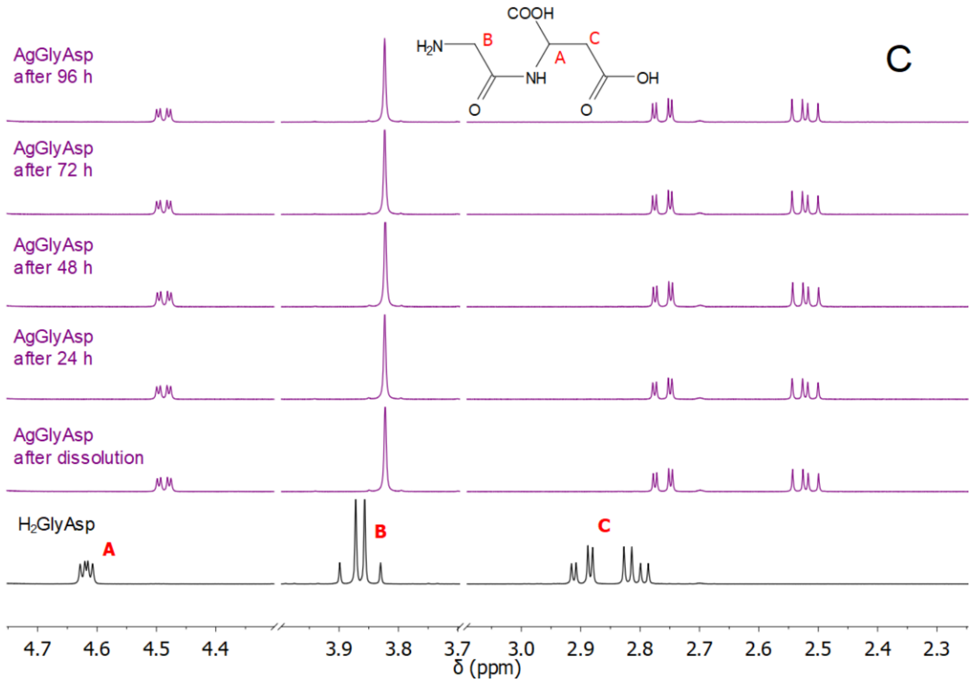
C
AgGlyAsp
after 96 h
AgGlyAsp
after 72 h
AgGlyAsp
after 48 h
AgGlyAsp
after 24 h
AgGlyAsp
after dissolution
H2GlyAsp
A
B
C
COOH
H2N
NH
OH
O
4.7
4.6
4.5
4.4
3.9
3.8
3.7
3.0
2.9
2.8
2.7
2.6
2.5
2.4
2.3
δ (ppm)

Figure S3. Spectroscopic titration in Tris-HCl solution for the AgGlyGly complex during titration with increasing amounts of CT DNA.

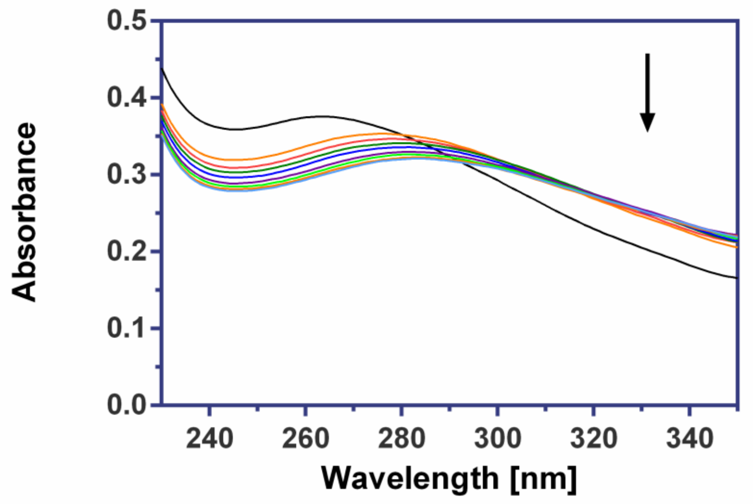

Figure S4. Spectroscopic titration in Tris-HCl solution for the AgGlyAla complex during titration with increasing amounts of CT DNA.

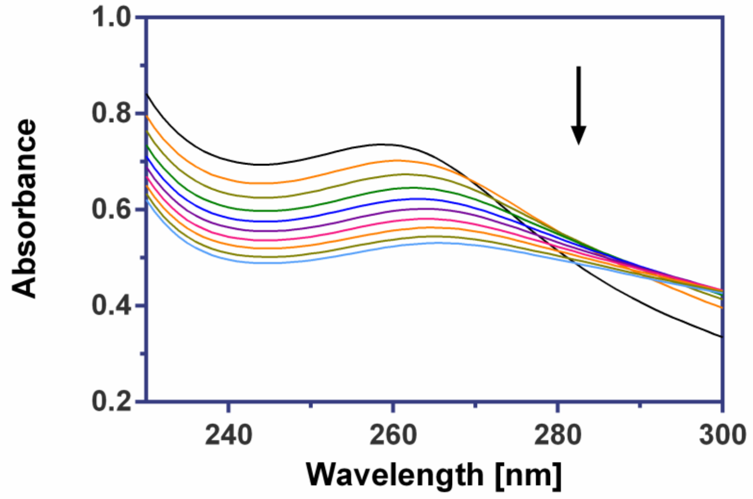

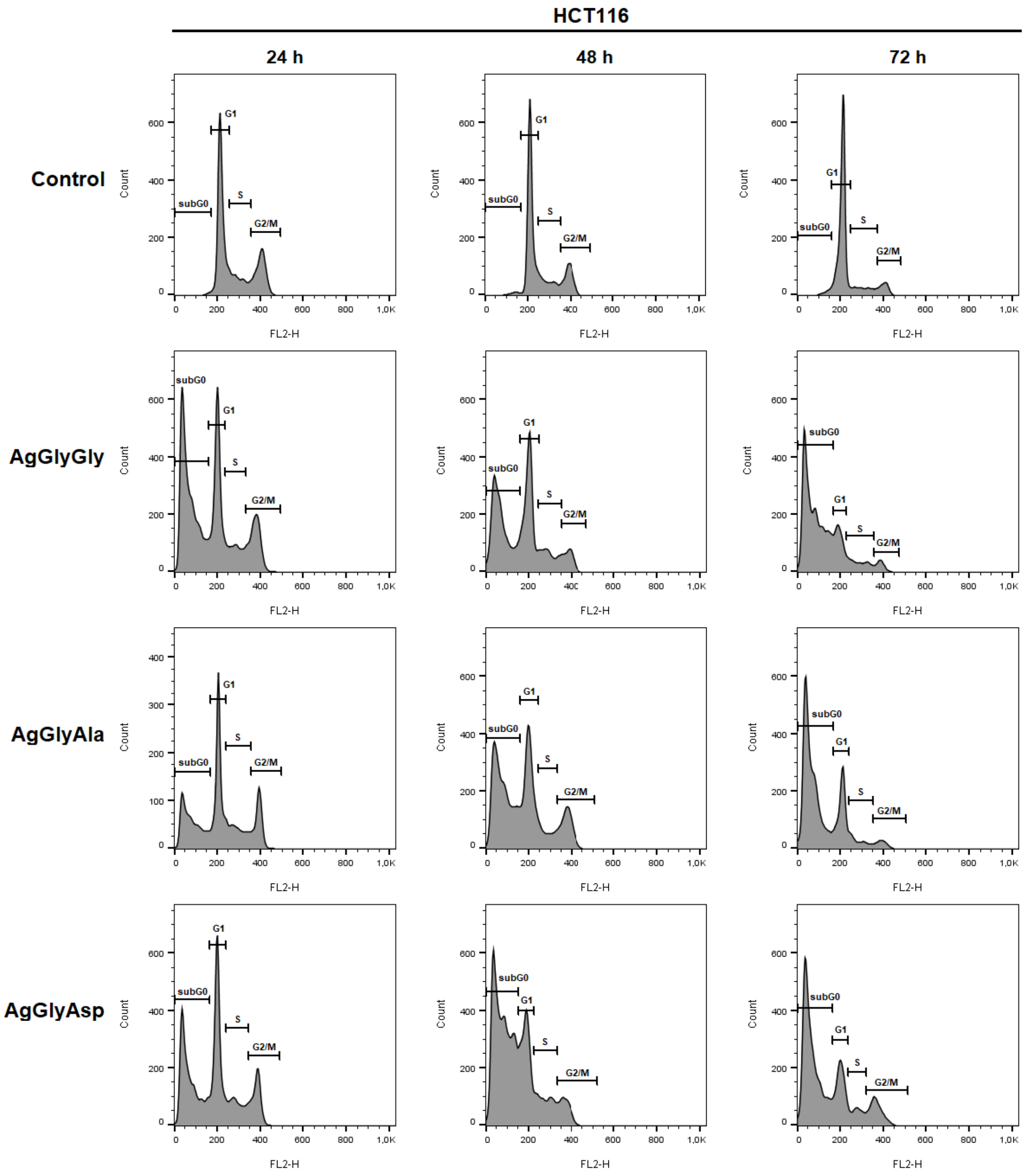

Figure S5. Histogram representation of cell cycle distribution in HCT116 cells treated with IC_{50} AgGlyGly, AgGlyAla, AgGlyAsp after 24 h, 48 h, and 72 h. Representative data of three independent experiments are presented.

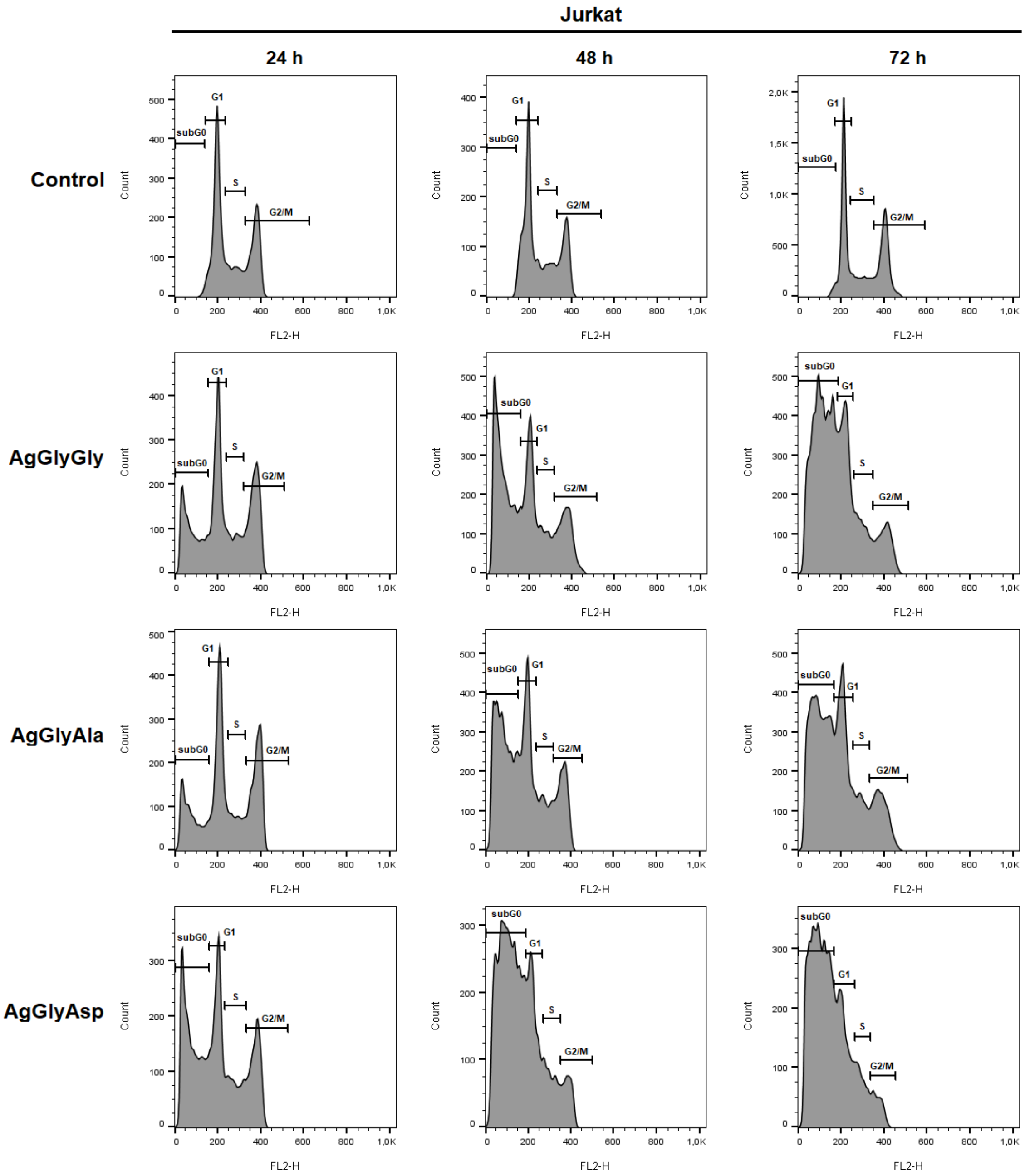

Figure S6 Histogram representation of cell cycle distribution in Jurkat cells treated with IC_{50} AgGlyGly, AgGlyAla, AgGlyAsp after 24 h, 48 h, and 72 h. Representative data of three independent experiments are presented.

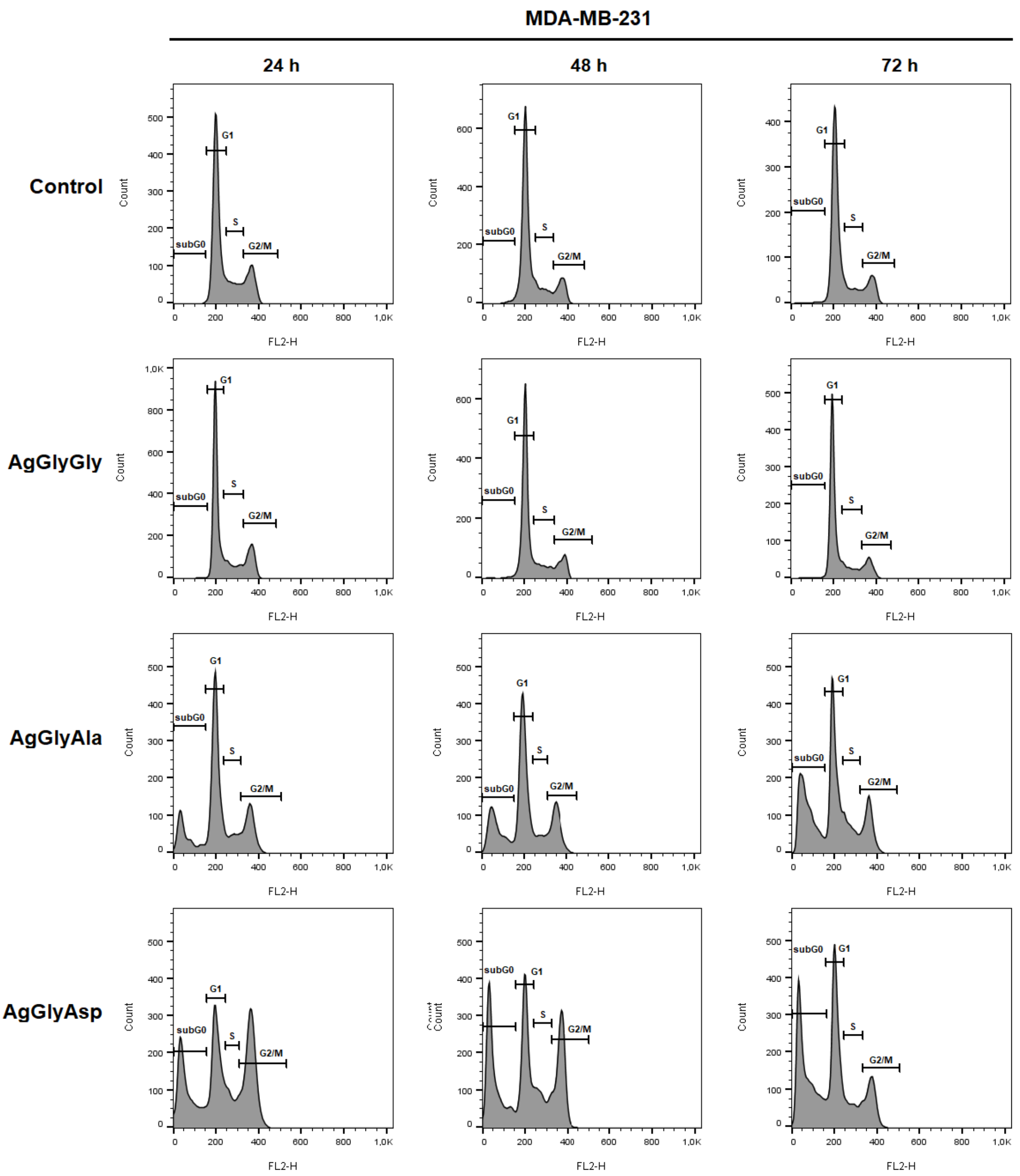

Figure S7. Histogram representation of cell cycle distribution in MDA-MB-231 cells treated with IC_{50} AgGlyGly, AgGlyAla, AgGlyAsp after 24 h, 48 h, and 72 h. Representative data of three independent experiments are presented.

Figure S8. Mass spectra of the aqueous solution of AgGlyGly complex measured in positive mode.

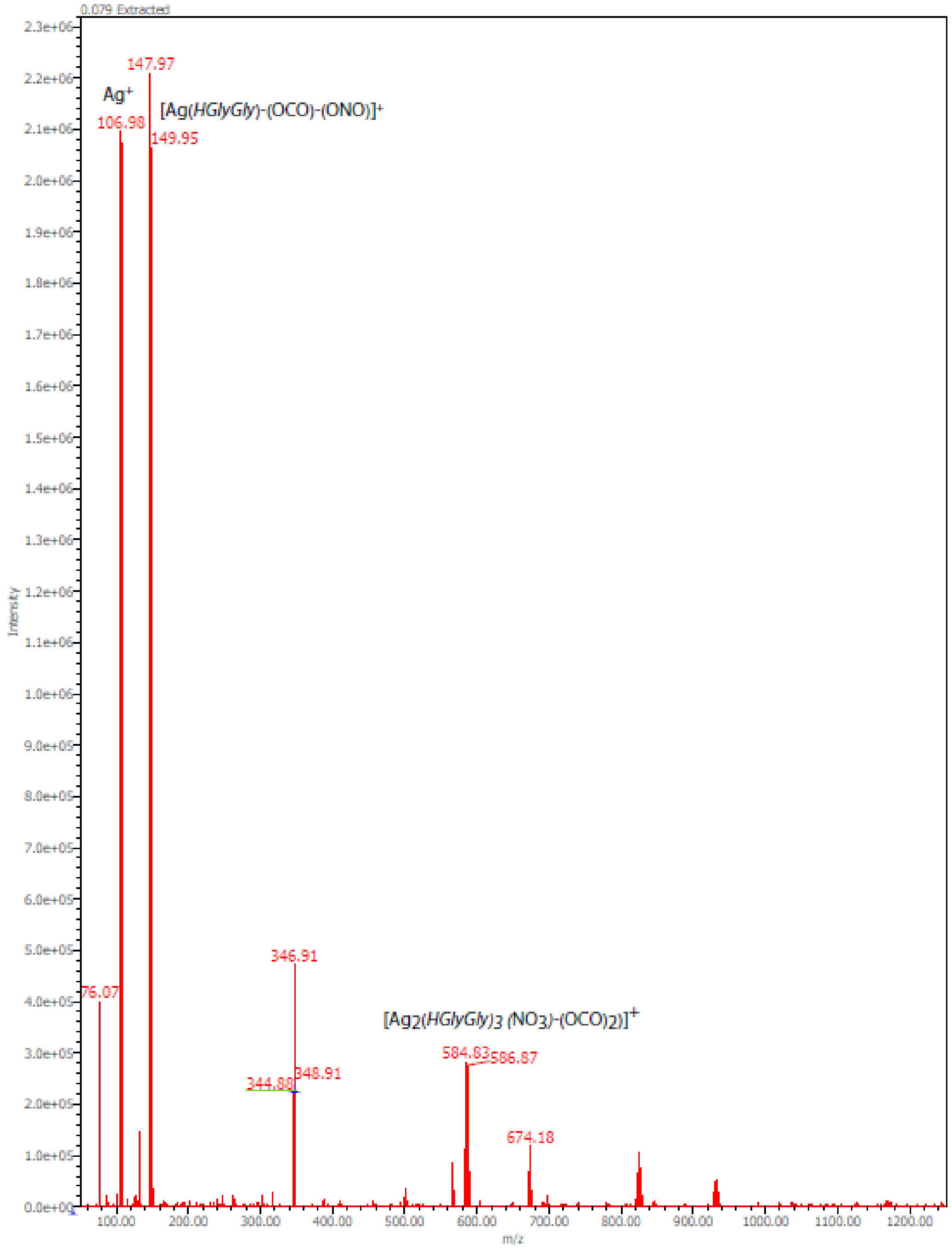

Figure S9. Mass spectra of the aqueous solution of AgGlyAla complex measured in positive mode.

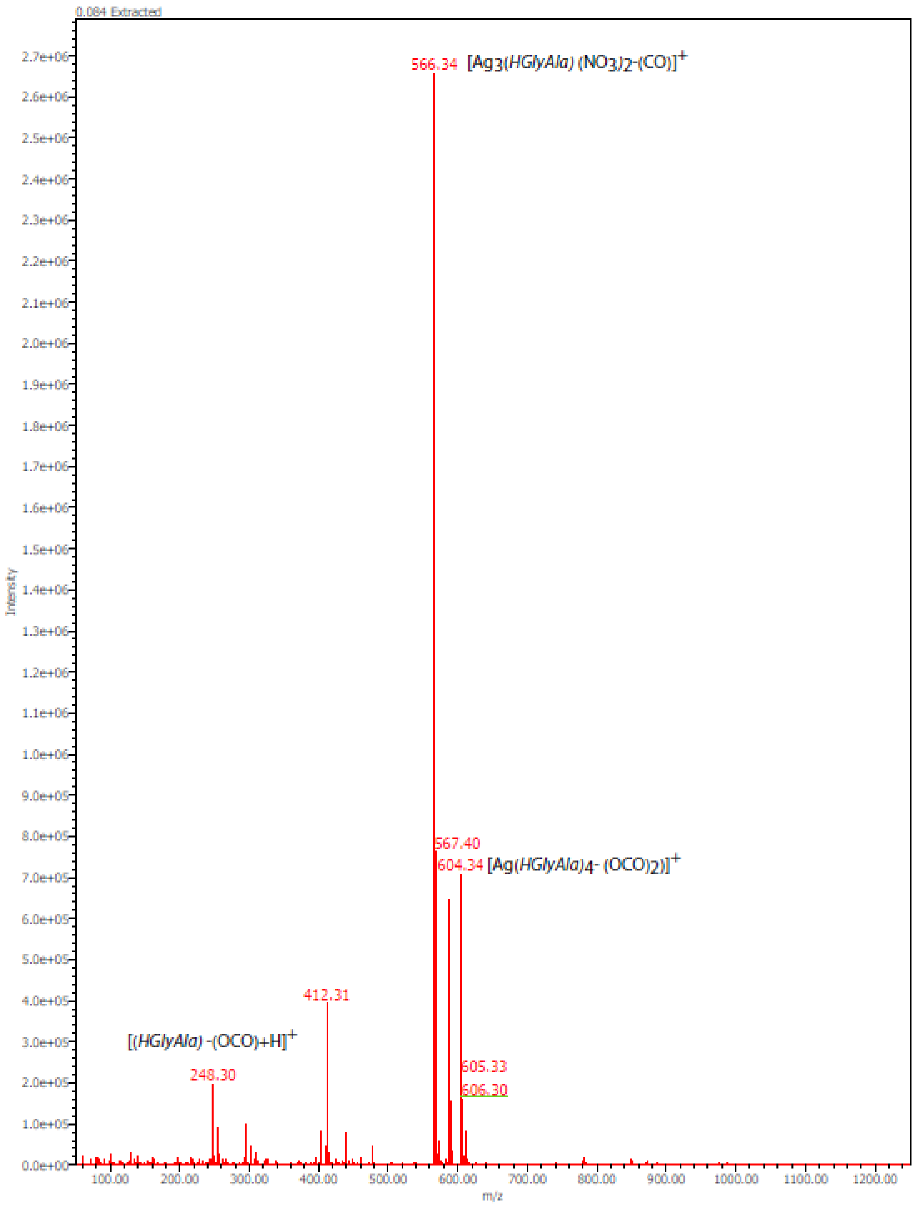

Figure S10. Mass spectra of the methanolic solution of AgGlyAsp complex measured in positive mode.

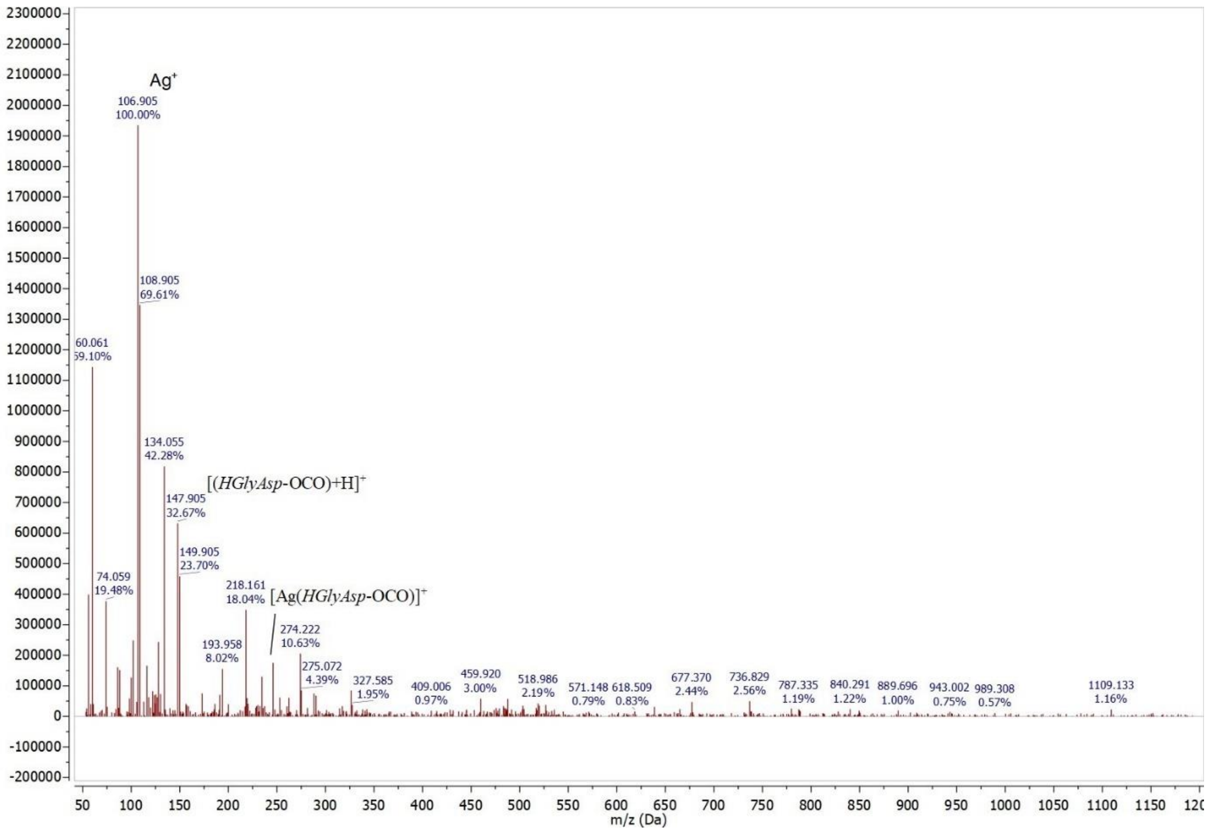

Table S1. Crystal data and structure refinement summary for complex AgGlyGly

Formula	$[Ag(HGlyGly)]_n(NO_3)_n$
Acronym	AgGlyGly
Empirical formula	$C_4 H_8 Ag N_3 O_6$
Formula weight	301.99
Crystal parameters	
Crystal size (mm)	0.981 x 0.580 x 0.194
Crystal system	Monoclinic
Space group	$P2_1/c$
Unit cell dimension	
a (Å)	4.9778 (7)
b (Å)	27.387 (4)
c (Å)	5.9015 (8)
α (°)	90
β (°)	94.022 (2)
γ (°)	90
V_c (Å^3)	802.6(2)
Molecules per cell, *Z*	4
D_{calc} (g.cm^{-3})	5.574
Measurement of intensity data	
Temperature (K)	120(2)
Wavelength (Å)	0.71073
θ range (°)	1.487 to 26.072
Reflections collected / unique	10247 / 1580 [R(int) = 0.0284]
Data	1580
F(000)	592
Indices limit	
H	-6 to 6
K	-33 to 33
L	-7 to 7
Refinement	
Refinement method	full-matrix least-squares on F^2
Parameters	133
Goodness-of-fit on F^2	1.256
R_1	0.035
wR_2	0.093
Maximum and minimum height in final difference synthesis (e.Å^3)	2.042 and -0.769

Table S2. Selected bond distances (Å) and angles (°) for AgGlyGly.

**Bond distances**			
Ag1—O2^{i}	2.198 (4)	C3—O3	1.229 (7)
Ag1—O1	2.206 (4)	C3—C4	1.521(7)
Ag1—O2^{ii}	2.595 (4)	C4—N2	1.488 (7)
Ag1—Ag^{i}	2.8928 (9)	C4—H4A	0.9900
O1—C1	1.252 (7)	C4—H4B	0.9900
C1—O2	1.265 (7)	N2—H2C	0.9100
C1—C2	1.518 (7)	N2—H2D	0.9100
C2—N1	1.450 (7)	N2—H2E	0.9100
C2—H2A	0.9900	N3—O5	1.236 (6)
C2—H2B	0.9900	N3—O4	1.262 (6)
N1—C3	1.342 (7)	N3—O6	1.269 (6)
N1—H3N	0.88 (7)		

**Bond angles**			
O2^{i}—Ag1—O1	161.57 (15)	O2^{ii}—Ag1—Ag1^{i}	119.35 (9)
O2^{i}—Ag1—O2^{ii}	85.64 (14)	C1—O1—Ag1	116.0 (3)
O1—Ag1—O2^{ii}	97.66 (14)	O1—C1—O2	126.9 (5)
O2^{i}—Ag1—Ag1^{i}	74.58 (10)	O1—C1—C2	118.7 (5)
O1—Ag1—Ag1^{i}	88.14 (10)	O2—C1—C2	114.4 (5)
C1—O2—Ag1^{i}	132.9 (4)	N2 —C4—H4A	110.0
C1—O2—Ag1^{iii}	118.1 (3)	C3—C4—H4A	110.0
Ag1^{i}—O2—Ag1^{iii}	94.36 (14)	N2—C4—H4B	110.0
N1—C2—C1	112.7 (5)	C3—C4—H4B	110.0
N1—C2—H2A	109.0	H4A—C4—H4B	108.3
C1—C2—H2A	109.0	C4—N2—H2C	109.5
N1—C2—H2B	109.0	C4—N2—H2D	109.5
C1—C2—H2B	109.0	H2C—N2—H2D	109.5
H2A—C2—H2B	107.8	C4—N2—H2E	109.5
C3—N1—C2	120.6 (5)	H2C—N2—H2E	109.5
C3—N1—H3N	118 (4)	H2D—N2—H2E	109.5
C2—N1—H3N	120 (4)	O5—N3—O4	120.7 (5)
O3—C3—N1	123.7 (5)	O5—N3—O6	120.6 (5)
O3—C3—C4	121.8 (5)	O4—N3—O6	118.7 (4)
N1—C3—C4	114.5 (5)		
N2—C4—C3	108.7 (4)		

Symmetry codes: (i) -x,-y,-z+3; (ii) x-1,y,z ; (iii) x+1,y,z

Table S3. Antibacterial activity of silver complex and free ligands (IC_{50}, MIC_{90}: µmol.l^{-1}).

	E. coli		***S. aureus***		***S. aureus* L12**		***S.epidermidis***	
	IC_{50}	MIC_{90}	IC_{50}	MIC_{90}	IC_{50}	MIC_{90}	IC_{50}	MIC_{90}
Gly	N		N		N		N	
Ala	N		N		N		N	
GlyGly	N		N		N		N	
GlyAla	N		N		N		N	
GlyAsp	N		N		N		N	
AgSD	1	4	1	2	1	4	2	5
**AgGly**	**3**	**5**	**3**	**5**	**5**	**10**	**5**	**10**
**AgAla**	**3**	**5**	**4**	**6**	**5**	**10**	**3**	**10**
AgGlyGly	6	10	12	22	15	38	10	20
**AgGlyAla**	**6**	**9**	**7**	**11**	**9**	**20**	**8**	**10**
AgGlyAsp	17	23	51	96	31	49	25	50

Note: N- not active IC_{50} and MIC_{90} values > 2 mmol.l^{-1}, Gly – glycine, Ala - alanine, SD-sulfadiazine

Table S4. Antifungal activity of silver complexes and free ligands.

	C. parapsilosis		***C. albicans***		***Rhizopus oryzae***		***A. alternata***		***A.flavus***		***M.gypseum***	
	**IC_{50}**	**MIC_{80}**	**IC_{50}**	**MIC_{80}**	**IC_{50}**	**MIC_{100}**	**IC_{50}**	**MIC_{100}**	**IC_{50}**	**MIC_{100}**	**IC_{50}**	**MIC_{100}**
Gly	N		N		N		N		N		N	
Ala	N		N		N		N		N		N	
GlyGly	N		N		N		N		N		N	
GlyAla	N		N		N		N		N		N	
GlyAsp	N		N		N		N		N		N	
AgSD	1	5	7	23	**3**	**10s**	**8**	**>100**	**46**	**89s**	**30**	**50s**
**AgGly**	**2,5**	**5**	**7**	**10**	**3**	**5s**	**5**	**10s**	**20**	**100s**	**100**	**>100**
**AgAla**	**25**	**50**	**7**	**10**	30	50s	>100	>100	NA		**100**	>100
AgGlyGly	30	70	70	100	30	50s	71	>100	NA		NA	
**AgGlyAla**	**30**	**60**	**50**	**87**	30	50s	42	>100	NA		**75**	100s
AgGlyAsp	50	100	73	100	73	100s	35	>100	NA		NA	

Note: N- not active IC_{50} and MIC values > 2 $mmol.l^{-1}$, NA – not active IC_{50} and MIC values >100$\mu mol.l^{-1}$, Gly – glycine, Ala - alanine, SD-sulfadiazine,s-fungistatic

Table S5. Cell cycle analysis of BLM cells after 24 h, 48 h, and 72 h incubation with dipeptides complexes.

**BLM**	**Sub-G0**	**G1**	**S**	**G2/M**
**CTRL 24 h**	2.2 ± 0.7	49.2 ± 0.1	18.6 ± 0.9	30.1 ± 0.3
**AgGlyGly**	17.3 ± 4.2 **	32.5 ± 2.8 **	14.1 ± 1.0 *	36.2 ± 1.3 *
**AgGlyAla**	13.5 ± 1.1 *	32.6 ± 0.1 **	17.9 ± 2.6	36.1 ± 0.9 *
**AgGlyAsp**	51.7 ± 0.5 ***	19.8 ± 2.7 ***	13.8 ± 3.0 *	14.8 ± 0.2 **
**CTRL 48 h**	2.0 ± 0.7	56.3 ± 0.6	20.5 ± 0.4	21.3 ± 0.4
**AgGlyGly**	18.8 ± 0.1 **	33.2 ± 0.9 **	15.3 ± 1.5 *	32.7 ± 2.4 **
**AgGlyAla**	13.7 ± 3.3 *	38.0 ± 0.5 **	19.5 ± 0.1	28.9 ± 2.7 *
**AgGlyAsp**	56.1 ± 0.9 ***	22.2 ± 2.8 ***	10.8 ± 2.5 *	10.9 ± 2.8 *
**CTRL 72 h**	1.6 ± 0.5	53.2 ± 2.0	22.3 ± 2.3	23.0 ± 0.9
**AgGlyGly**	22.8 ± 2.8 **	34.1 ± 2.9 **	16.0 ± 2.5 *	27.1 ± 2.1 *
**AgGlyAla**	13.8 ± 0.1 *	40.1 ± 1.6 **	18.8 ± 0.4	27.3 ± 1.3
**AgGlyAsp**	59.3 ± 3.4 ***	16.9 ± 3.5 ***	14.6 ± 0.9 *	9.4 ± 0.7 *

The data are presented from three independent experiments after 24 h, 48 h, and 72 h treatment as average percentage ± SD. Significantly different * $p < 0.05$, ** $p < 0.01$, *** $p < 0.001$ vs untreated cells (control).

Table S6. Cell cycle analysis of HCT116 cells after 24 h, 48 h, and 72 h incubation with dipeptides complexes.

HCT116	Sub-G0	G1	S	G2/M
**CTRL 24 h**	0.8 ± 0.2	50.9 ± 3.6	18.5 ± 0.7	29.8 ± 3.2
**AgGlyGly**	44.3 ± 2.0 ***	26.3 ± 1.2 **	13.6 ± 2.4 *	15.8 ± 0.8 *
**AgGlyAla**	32.4 ± 0.9 **	37.8 ± 2.4 **	18.9 ± 0.8	10.9 ± 2.5 *
**AgGlyAsp**	37.2 ± 1.3 **	29.8 ± 3.4 **	16.3 ± 2.3	16.8 ± 2.4 *
**CTRL 48 h**	3.7 ± 0.8	56.2 ± 3.9	20.0 ± 2.4	20.3 ± 0.7
**AgGlyGly**	50.8 ± 2.0 ***	24.8 ± 1.1 **	13.7 ± 0.7 *	10.8 ± 2.5 *
**AgGlyAla**	46.2 ± 0.4 ***	32.9 ± 1.2 **	7.0 ± 0.3 *	14.0 ± 0.4 *
**AgGlyAsp**	57.1 ± 0.4 ***	21.0 ± 1.7 ***	10.3 ± 1.3 *	11.7 ± 3.4 *
**CTRL 72 h**	3.5 ± 0.1	69.4 ± 0.9	13.5 ± 0.5	13.6 ± 0.4
**AgGlyGly**	62.9 ± 5.3 ***	20.3 ± 2.9 ***	9.8 ± 0.1 *	7.1 ± 2.3 *
**AgGlyAla**	56.8 ± 4.3 ***	27.6 ± 2.6 ***	8.1 ± 1.2 *	7.6 ± 1.7 *
**AgGlyAsp**	62.7 ± 1.6 ***	20.3 ± 0.3 ***	7.7 ± 0.1 *	9.3 ± 2.0 *

The data are presented from three independent experiments after 24 h, 48 h, and 72 h treatment as average percentage ± SD. Significantly different * $p < 0.05$, ** $p < 0.01$, *** $p < 0.001$ vs untreated cells (control).

Table S7. Cell cycle analysis of Jurkat cells after 24 h, 48 h, and 72 h incubation with dipeptides complexes.

**Jurkat**	**Sub-G0**	**G1**	**S**	**G2/M**
**CTRL 24 h**	1.0 ± 0.1	50.1 ± 0.2	16.2 ± 1.3	32.8 ± 1.5
**AgGlyGly**	25.9 ± 0.3 **	34.5 ± 0.8 **	12.8 ± 0.1 *	26.9 ± 0.4 *
**AgGlyAla**	20.4 ± 1.9 **	36.1 ± 0.4 **	12.7 ± 0.1 *	30.9 ± 1.5
**AgGlyAsp**	46.9 ± 3.2 ***	23.6 ± 1.9 **	14.0 ± 0.6	15.5 ± 2.7 *
**CTRL 48 h**	0.7 ± 0.4	53.0 ± 1.2	22.1 ± 1.5	24.3 ± 0.6
**AgGlyGly**	46.1 ± 0.1 ***	25.6 ± 0.8 **	11.8 ± 0.5 *	16.6 ± 1.3 *
**AgGlyAla**	39.7 ± 3.7 ***	29.9 ± 1.3 **	12.6 ± 0.5 *	17.9 ± 1.8 *
**AgGlyAsp**	57.4 ± 4.2 ***	24.0 ± 3.4 **	12.4 ± 1.1 *	6.3 ± 0.3 *
**CTRL 72 h**	2.3 ± 0.3	46.8 ± 2.9	16.6 ± 1.2	34.4 ± 1.3
**AgGlyGly**	57.0 ± 0.6 ***	23.0 ± 0.1 **	11.1 ± 0.8 *	8.9 ± 0.1 **
**AgGlyAla**	53.2 ± 3.6 ***	24.4 ± 2.3 **	12.1 ± 1.8 *	10.3 ± 3.1 **
**AgGlyAsp**	59.4 ± 1.8 ***	23.8 ± 0.1 **	11.1 ± 1.8 *	5.8 ± 0.2 **

The data are presented from three independent experiments after 24 h, 48 h, and 72 h treatment as average percentage ± SD. Significantly different * $p < 0.05$, ** $p < 0.01$, *** $p < 0.001$ vs untreated cells (control).

Table S8. Cell cycle analysis of MDA-MB-231 cells after 24 h, 48 h, and 72 h incubation with dipeptides complexes.

MDA-MB-231	Sub-G0	G1	S	G2/M
**CTRL 24 h**	1.0 ± 0.6	66.4 ± 1.3	14.1 ± 1.4	18.6 ± 0.5
**AgGlyGly**	1.8 ± 0.4	62.3 ± 0.2	16.6 ± 0.3	19.3 ± 0.9
**AgGlyAla**	17.7 ± 3.0 *	48.3 ± 0.2 **	11.2 ± 2.0	22.8 ± 2.5 *
**AgGlyAsp**	24.7 ± 2.0 **	37.2 ± 4.2 **	7.6 ± 2.5 *	30.6 ± 2.4 *
**CTRL 48 h**	1.9 ± 0.4	68.5 ± 1.7	14.4 ± 1.3	15.3 ± 0.7
**AgGlyGly**	2.1 ± 0.2	68.4 ± 0.1	14.7 ± 0.2	14.9 ± 0.3
**AgGlyAla**	21.1 ± 2.5 **	48.5 ± 1.8 **	11.1 ± 0.7	19.4 ± 0.1 *
**AgGlyAsp**	35.8 ± 2.2 **	27.8 ± 0.8 ***	10.6 ± 1.9 *	25.8 ± 0.5 *
**CTRL 72 h**	2.2 ± 0.3	69.2 ± 0.1	13.8 ± 0.9	14.9 ± 0.5
**AgGlyGly**	2.3 ± 0.1	68.5 ± 0.1	14.4 ± 0.1	14.9 ± 0.1
**AgGlyAla**	40.5 ± 3.3 ***	35.0 ± 2.3 **	10.9 ± 1.3	13.7 ± 1.8
**AgGlyAsp**	55.9 ± 0.5 ***	27.4 ± 2.3 ***	7.1 ± 2.4 *	9.6 ± 1.2 *

The data are presented from three independent experiments after 24 h, 48 h, and 72 h treatment as average percentage ± SD. Significantly different * $p < 0.05$, ** $p < 0.01$, *** $p < 0.001$ vs untreated cells (control).
